# The Use of Ultrasound for Preventing Honey Crystallization

**DOI:** 10.3390/foods10040773

**Published:** 2021-04-04

**Authors:** Laura Agripina Scripcă, Sonia Amariei

**Affiliations:** Faculty of Food Engineering, Stefan cel Mare University of Suceava, 720229 Suceava, Romania; sonia@usm.ro

**Keywords:** ultrasound treatment, chemical composition of honey, textural and rheological parameters

## Abstract

The aim of this study was to evaluate the effect of ultrasound treatment for preventing honey crystallization on the physicochemical and microbiological properties of unifloral honey and polyfloral honey. Honey samples without any treatment were used as a control group for comparison. The effect of applying ultrasound treatment was evaluated by studying over time the tendency of crystallization, the rheological properties of honey and chemical and microbiological properties. The parameters analyzed for the two groups of samples (treated and untreated with ultrasound), which did not vary or had small variations during the research were water content, acidity, water activity, glucose, fructose, sucrose, glucose/water ratio, glucose/fructose ratio. The crystallization process was installed in the control samples from the first month of the study, and much later in the treated samples. The color of the untreated samples varied considerably, and the color of the treated ones remained stable or slightly varied. For the control samples, the smallest variation in hydroxymethylfurfural (HMF) concentration was in raspberry honey (5%), and the most significant variation was in honeydew honey (30%). For the treated samples, the largest variation of this parameter was found in tillia honey (127%), and the smallest variation was in rapeseed honey (26%). The microbiological quality was higher for the treated samples. In the ultrasound-treated samples of acacia honey, honeydew honey and grassland honey, yeasts, molds or standard plate counts (SPCs) were undetectable. For control samples, SPC values were <10–50 cfu/g. Ultrasound-treated samples maintained their SPC parameter levels or were thus reduced (<10–20 cfu/g). Yeasts and molds were undetectable or had value between <10 and 10 cfu/g. The yeasts and the molds ranged in the control samples between <10 and 40 cfu/g.

## 1. Introduction

Honey is a natural sweet substance produced by *Apis mellifera* bees, according to Directive 2001/110 EC [1]. The chemical composition of honey is characterized mainly by the high percentage of sugars (approximately 80%), glucose and fructose being the main compounds [2]. The other 20% is water, minerals, vitamins, organic acids, phenolic compounds, enzymes [3,4].

The ratio of the main sugars, glucose and fructose, is important in the evaluation of the crystallization tendency of honey [5]. Glucose tends to crystallize spontaneously due to its low solubility in water [6]. High glucose content and high glucose/fructose ratio determine the faster crystallization of honey (for example, rapeseed, honeydew, polyfloral honey), and low values of glucose content and glucose/fructose ratio cause slower crystallization of honey (acacia, raspberry honey) [7,8]. The glucose/water ratio is considered another indicator of honey crystallization [9]. The value of the glucose/water ratio below 1.7 indicates that the honey crystallizes slowly, and a value higher than 2.0 indicates rapid crystallization [10,11]. The ability of honey to crystallize is different depending on its assortment and source. The appearance of crystallization in honey is an undesirable process [12] because for consumers this process is a reason to refuse the consumption of the product [13].

The prevention of honey crystallization or decrystallization is achieved by thermal processing [14]. Heat treatment can increase the hydroxymethylfurfural level, even beyond the legal limit [14]. Due to heat treatments, changes in enzymatic activity (decrease) and antioxidants (increase) occur. Color changes can also occur, respectively, browning of treated honey [14]. Another method used to liquefy honey or prevent the installation of this process is to treat honey with ultrasound [15]. In the food industry, ultrasonic treatment can have, among other things, the effect of improving crystallization and changing the texture of products [15]. This treatment acts on the yeasts it destroys, inhibits the crystallization of honey and improves its appearance [16]. Ultrasound influences the growth capacity of cells that resist treatment, reducing the risk of honey fermentation [16]. Ultrasonic and conventional heating treatments were applied to honey and the results showed that both treatments had a significant effect on microbial reduction and color, pH, diastase activity and hydroxymethylfurfural (HMF) concentration changed slightly [17]. Simultaneously, the ultrasonic samples have improved the content of total polyphenols, total flavonoids and antioxidant capacity, which makes ultrasonic treatment an alternative way of preserving honey and maintaining its quality [17]. The thermal process using ultrasound as a pretreatment, has improved several properties of honeydew honey, such as antioxidant, antimicrobial and polyphenol content [18]. Ultrasonic treatment had a different effect on the properties of honey samples from Northeast India, decreasing total polyphenol content [19]. The application of ultrasound treatment resulted in increased flavonoid content and exceeding this time caused them to decrease [19]. The ultrasound treatment did not affect the concentration of HMF and the diastatic activity of sunflower honey; it even improved the diastatic activity [20]. The concentration of HMF varied differently when applying different ultrasound treatments. The ultrasound treatment time was important for increasing HMF concentration [21]. At the same time, the diastatic activity, HMF content, the polyphenols and flavonoid content of the ultrasound-treated samples varied differently, depending on their botanical source [22]. The effect of ultrasound on the quality of honey was studied by comparing the ultrasonic treatment applied for different times, amplitudes and sample volume, with heat treatment in different temperature conditions. It was observed that treating a volume of 60 mL of honey for 8 min at an amplitude of 60% did not decrease diastase activity and did not increase HMF concentration while other physicochemical parameters such as water content, pH and color were less affected. From a microbiological viewpoint, this treatment has reduced the number of aerobic mesophilic bacteria, which makes it an alternative nonthermal technique for processing honey [23]. Ultrasonic treatment for liquefaction of crystallized honey is an advantageous alternative to conventional heat treatment. After the ultrasound treatment, the changes of the physicochemical parameters are followed and it is desired to improve or maintain these parameters of quality [24]. The use of ultrasound in the case of unifloral honey has shown that it affects increased HMF content, antioxidant activity and polyphenol content after storage [25]. The ultrasonication of honey does not cause significant changes in physical properties of these [26]. Ultrasonic treatment of longan honey under different conditions of temperature, time and amplitude improved the bioactive compounds of the samples without influencing their nutritional value [27]. Ultrasonic treatment can replace the conventional heat treatment for honey with multiple benefits including the reduction and elimination of formed crystals [28,29]. Applying an ultrasound treatment produces a reduction in the number and size of honey crystals [30]. 

The aim of this study was to determine the effects of applying ultrasound treatment on the crystallization tendency of honey and its influence on the quality parameters of honey, such as water content, acidity, HMF, diastase activity, color and texture. At the same time, the aim of this research was to identify the effects of the treatment applied on the microorganisms present in honey and on the microbial growth.

## 2. Materials and Methods

### 2.1. Materials

The honey assortments analyzed were acacia honey, raspberry honey, tillia honey, polyfloral honey, rapeseed (rape) honey, honeydew honey and grassland honey and were purchased from authorized local beekeepers from North Romania (commercial company—Apicola Suceava, Suceava, Romania). The botanical origin of honey was guaranteed by the company from which the samples were purchased. The samples were from the honey production of 2019. Control samples were samples of fresh honey that were not subjected to ultrasound treatment and were weighed (500 g each). They were stored at 18–20 °C, in glass jars, in a dark environment and they were analyzed at one month, three months, five months, seven months and nine months.

The reagents used were analytically pure. Double deionized water (18 MΩ cm resistivity) was produced by a water purification system (Thermofisher, Germany) and it was used in all solutions. Sugar standards as d (+) glucose, d (−) fructose and d (+) sucrose were purchased from Santa Cruz Biotechnology Inc. (Dallas, TX, USA). Phenolphthalein indicator and starch solutions were purchased from Lachner (Neratovice, Czech Republic). Sigma Aldrich (Steinhein, Germany) has provided sodium chloride solution, para-toluidine, iodine solution, barbituric acid, acetic acid, agar plates, potato-dextrose agar (PDA), Sabouround agar, and Fluka (Seelze, Germany) has provided a sodium carbonate. Sodium chloride solution and sodium hydroxide were purchased from Sigma (St. Louis, Missouri, USA). Acetonitrile was provided by Scharlau (Barcelona, Spain) and agar Deoxycholate Citrate Lactose (ADCL) was provided by todylaboratories.

### 2.2. Methods

Each parameter investigated for each honey sample was determined in triplicate.

#### 2.2.1. Ultrasound Treatment

The fresh honey was weighed (500 g each) in glass jars and subjected to ultrasonic treatment. The ultrasonic treatment was applied to honey samples, using an ultrasonic bath (BRANSON 3510, Danbury, CT, USA) fitted with thermostat for temperature control. The temperature was 20 °C, the time was 1 h and the frequency was 42 kHz [21]. The initial temperature was 20 °C and the final temperature (at the end of the ultrasound treatment) was 25 °C. The temperature was controlled by dividing the treatment time (1 h) into three rounds of 20 min of treatment, with a 20 min pause between each round. The treated samples were stored at 18–20 °C in a dark environment and they were analyzed and compared to the control samples, from a physicochemical and microbiological viewpoint, after the application of the treatment at three months, five months, seven months and nine months. 

#### 2.2.2. Water Content

The water content of honey samples was determined in triplicate, using Refractometer RE40 (Mettler Toledo, OH, USA), by measuring the refractive index at 20 °C. This analysis was based on the direct correlation between the refractive index and the solids content of a sample, and it was necessary to calculate water content according to the Harmonized Methods of the International Honey Commission [31].

#### 2.2.3. Sugars

Glucose, fructose and sucrose were analyzed according to the Harmonized Methods [31], by HPLC 10 AD VP (Shimadzu Corp., Kyoto, Japan), with a refractive detector [32].

#### 2.2.4. Acidity

The acidity was determined by the classical titration method with 0.1 mol/L NaOH solution, in the presence of phenolphthalein as indicator. The results were expressed in mEq/kg of honey [31].

#### 2.2.5. Diastase Activity and Hydroxymethylfurfural Concentration

The analysis of diastase activity was performed by determining the activity of amylase. The diastase index is defined as the number of milliliters of 1% starch solution which has been processed in dextrin for 1 h at 45 °C at an optimum pH of amylase containing 1 g of honey. Diastase activity was determined by the Schade procedure.

Diastase activity and hydroxymethylfurfural were determined according to the methods of the STAS 784–2009 [33]. Hydroxymethylfurfural (HMF) was determined by the Winkler method. HMF concentration was determined using a Perkin Elmer UV-VIS LAMBDA EZ201, at a wavelength of 550 nm, equipped with cuvettes with a layer of 1 cm thickness. The color intensity of the red complex formed by HMF with barbituric acid in the presence of para-toluidine is proportional to the concentration of HMF.

#### 2.2.6. Water Activity

Water activity was determined with the Aqua Lab 4Tev Duo (Pullman, WA, USA).

#### 2.2.7. Color Measurement

Color was determined using a Chroma Meter Konika Minolta CR-410. CIE L* a* b* color of the samples was determined using Chroma Meter CR-410 (Konica Minolta, Tokyo, Japan). In CIE L* a * b * color space, the three values of the coordinates are as follows: L for lightness has ranged from zero to 100 (from black to white), a* has ranged from −60 to +60 (from green to red) and b* has a range from −60 to +60 (from blue to yellow). Chroma Meter was calibrated with reference white porcelain tile (L* = 97.63; a* = 0.31; b* = 4.63) before the determination [9]. The illuminant used was D65 [34].

Additionally, the Chroma (relative saturation) coordinates and the Hue angle are calculated as follows from the Equations (1) and (2), [35]:

(1) $$Chroma=\sqrt{a^{2}+b^{2},}$$

(2) Hue Angle(°)=arctan(b∗/a∗),

Yellow Index describes the change in color of a control sample from clear or white to yellow and it is calculated with Equation (3), [36]:

(3) Yellow Index=142.86×b∗/L∗,

#### 2.2.8. Texture Profile Analysis

Texture profile analysis (TPA) was determined with a Mark 10 Texture Analyzer (Mark 10 ESM301 Corporation, Copiague, NY, USA) equipped with a 50 mm disk probe. The diameter of the balloon was 70 mm. The TPA was performed at a constant speed of 150 mm/min, up to a depth of 12.5 mm and the honey column was 25 mm. The TPA analysis has two loading cycles: the first cycle is the compression cycle and is performed at a degree of deformation between 30 and 80% and the second represents the discharge cycle and occurs at the same speed as the first cycle. Textural parameters such as hardness, springiness, cohesiveness, adhesiveness, viscosity, chewiness and gumminess were measured at 20 ± 1 °C.

#### 2.2.9. Microbiological Analysis

The microbiological analysis was performed according to ISO quality standards—Microbiology of food and animal feeding stuffs [32]. Sample preparation was performed by mixing 10 g of each honey sample with 90 mL of peptone saline (8.5 g/L) to obtain the initial dilution. The mixture was then used for the other dilutions. All colonies that appeared after incubation were counted. The microbial number was expressed in colony forming units/gram of honey (cfu/g).

#### 2.2.10. Crystals Analysis

Observation of the content of crystals, the presence or absence of crystals in ultrasound-treated and untreated honey samples was performed with a stereomicroscope (Microscope Optika SZ A1, Italy) at a magnification of 40X. A 1 mL aliquot of the honey sample was placed on a lamella fixed with a sample holder.

#### 2.2.11. Statistical Analysis

The results were analyzed by principal component analysis (PCA), Pearson correlation and descriptive analyses with XLSTAT software (2019, trial version, Yaphank, NY, USA). PCA evaluated the correlations between the honey types and the sugars, color parameters and texture parameters, analyzed the variation and extracted the main components. Pearson correlations between the variables were calculated using Pearson’s coefficient (a *p*-value < 0.05 was considered statistically significant).

## 3. Results and Discussion

Physicochemical and microbiological parameters of seven types of honey (acacia, raspberry, tillia, polyfloral, rapeseed, grassland and honeydew) were investigated. Table 1 and Table 2 and Supplemental Material Tables S1–S3 present the quality parameters (water content, sugars, acidity, diastase activity, HMF, water activity, color, texture, yeast, molds, number of coliforms, number of standard counts, *Bacillus cereus*) of the honey assortments analyzed over nine months and the changes that occur during the research on the ultrasound-treated samples, compared to the control (untreated) sample.

### 3.1. Water Content

Monitoring and control of the water content of honey are important because this parameter significantly influences the crystallization process [12]. The G/W ratio is an important indicator of the tendency and degree of crystallization of honey, but it is not the only one [37]. According to Codex Alimentarius [38], water content should not exceed 20% in honey, to avoid the fermentation process during storage caused by osmotolerant yeasts [39]. The water content also influences the fluidity of honey and some texture properties such as adhesiveness, water activity, microbiological properties and microbial growth [40]. The water content of honey decreases during storage [41].

Water activity is used to determine the stability of honey. A value of water activity below 0.60 indicates good stability of honey and it does not allow microbial growth [42]. A water activity value higher than 0.60 can favor the growth of xerophilic molds, osmotolerant yeasts and halophilic bacteria [43]. Water activity values measured in this study are consistent with results reported by other scientists [44].

The values of the water content of the seven types of honey analyzed and treated were normal and did not exceed the maximum limit of 20%, which means that they complied with legal regulations established by the Codex Alimentarius [38]. The water content of the analyzed samples varied relatively slightly (had minor variations) during the nine months of research. The lowest water content was found in the ultrasound-treated honey sample, with 15.8% in the ninth month of the research, and the highest content of 17.6% was found in acacia honey.

The variation of water content for the untreated samples was between 0.5% and 1.23%. The smallest variation was registered for acacia, tillia and polyfloral honey, and the largest for honeydew honey. In the case of the treated samples, the water content was constant for grassland honey and had a 1.85% variation in raspberry honey and 0.5% variation in tillia honey. For the control sample of acacia honey, a G/W ratio was 1.54. This value was the lowest of the analyzed samples and the highest value of this parameter was registered for rapeseed honey, respectively 2.84.

The water activity of the analyzed samples changed little after the application of the ultrasound treatment and within 9 months of storage. The highest value of water activity was in raspberry and in honeydew honey, respectively, equal to 0.62, and the lowest value was 0.52, found in tillia honey.

These results confirm that a G/W ratio less than 1.3 would cause a partial and slow crystallization, respectively; the crystallization is complete and fast when this ratio is equal to or greater than 2.1 [45]. The decrease in the water content of the samples during ultrasound treatment can be due to the mechanical effects of breaking the microbubbles, which increases the mass transfer; simultaneously, the honey tends to occupy the previous place occupied by the microbubbles, resulting in shear forces.

Marghitaș et al. [46] observed in Romanian honey similar results of water content, reporting values between 16.6% and 20%, while Abramovic et al. [47] reported values of water content between 13.4% and 18.6% for floral honey from Slovenia. The water content of Argentine honey was between 17.9% and 18.5%, as reported by Maldonado et al. [48] and had values between 13.8% and 20%, as reported by Chirife et al. [49].

El Sohaimy et al. [50] reported G/W ratio values of 0.72 for Kashmiri honey, 1.56 for Yemeni honey, 1.38 for Saudi honey and 1.45 for Egyptian honey and Chua et al. [51] reported in their study that the G/W ratio for the four types of honey analyzed ranged from 1.03 for forest honey to 1.89 for acacia honey. The results obtained in our study are in accordance with the results reported by these scientists.

Water activity is directly correlated with the water content of honey and it had no significant variations, which indicated the stability of all honey samples in time. Similar results were obtained by Mădaş et al. [52] for acacia honey and by Łuczycka et al. [53] for three types of Polish honey: rapeseed honey, polyfloral and goldenrod honey. The average values of water activity in Slovenian honeydew honey and polyfloral honey were similar to those in our study (0.52–0.66) [54].

### 3.2. Sugars

The main sugars (glucose, fructose and sucrose) in the treated and the untreated samples were analyzed for a period of nine months. The results obtained are shown in Table 1.

The sugar content evaluation is important because it can give clues about the purity of honey or its authenticity [55]. Fructose and glucose are major sugars in honey, representing approximately 30–40% of honey components. Sucrose is naturally present in honey in a much smaller amount, ideally under 5%. This percentage varies depending on the botanical origin [56]. A high concentration of sucrose in honey indicates that honey was harvested too early before sugar was completely converted to glucose and fructose under the action of invertase or due to bees being fed with sugar syrup in spring [57]. Higher glucose content in honey indicates a faster crystallization and implicitly higher fructose content, and slower crystallization. The ratio between the two sugars is ideally between 0.9 and 1.35. The fructose to glucose ratio less than 1.0 indicates a rapid crystallization of honey and a ratio above 1.0 indicates a slow honey crystallization [50].

Subjecting honey samples to ultrasound treatment does not influence the sugar levels in honey. The lowest content of glucose was found in acacia honey, −27.34 g/100 g honey, and the highest one in rapeseed honey, −46.30 g/100 g honey. This indicates that acacia honey will crystallize slowly and rapeseed honey will crystallize faster. In acacia honey, the highest fructose content is 43.64 g/100 g and rapeseed honey has the lowest content of fructose, −23.60 g/100 g. The highest level of sucrose concentration was in ultrasound-treated samples of raspberry honey with 2.10 g/100 g and the lowest level of sucrose in honeydew honey with 0.87 g/100 g. The G/F ratio was smaller than 1.0 for the analyzed acacia, raspberry and grassland honey. These kinds of honey have a smaller tendency to crystallize. A higher ratio value of 0.93 was found for grassland honey and the lowest value of 0.62 was found for acacia honey. Tillia, polyfloral, rapeseed and honeydew honey have a G/F ratio > 1.0, which means that they will crystallize faster. Rapeseed honey has the highest value of this parameter, indicating rapid crystallization.

It was found that a G/F ratio of 2.5/1 is the critical ratio in the crystallization of honey [8]. A higher G/W ratio of 2.84 was found for rapeseed honey, and a lower ratio was found for acacia honey (1.54).

Homrani et al. [58] found results similar to those obtained by us, as glucose contents in Algerian honey harvested from several areas was between 23.1 g/100 g and 34.1 g/100 g, and fructose was between 33 g/100 g and 44.4 g/100 g. In another study, Tomczyk et al. [59] reported results mostly different from those presented in this study; in the sugar content of five types of honey, polyfloral, tillia, forest, rapeseed and acacia, the lowest glucose content was 28.43 ± 1.37 g/100 g in tillia honey and the highest was in rapeseed honey, 32.92 ± 2.80 g/100 g. The lowest fructose content was 34.67 ± 0.31 g/100 g in forest honey, the highest fructose content was 39.23 ± 3.08 g/100 g found in polyfloral honey and the G/F ratio was between 0.77 and 0.90 for tillia honey and rapeseed honey, respectively. Values of glucose content in honey from different botanical origins analyzed by Zielinska et al. [60] varied between 17.0 g/100 g and 36.3 g/100 g for lime honey and polyfloral honey, respectively, and fructose content varied from 28.3 g/100 g for lime honey to 53.8 g/100 g for buckwheat honey.

### 3.3. Acidity

The acidity of honey is important because this parameter can influence the stability, shelf life and texture of honey [19]. The level of free acidity can indicate the degree of freshness of honey [61]. High acidity indicates the existence of fermentative processes of sugars present in honey and organic acid content produced by the oxidation of glucose under the action of glucose oxidase [62]. These compounds are directly related to sensory properties such as the honey flavor.

In this study, the acidity values were normal; they did not exceed 50 meq/kg, the maximum legal limit. Ultrasonic treatment applied to seven types of honey influenced the acidity of the samples. The acidity of the control samples increased from the first to the last month of the study and the acidity values of the ultrasound-treated samples decreased during the study. The acidity of the control samples had the largest variation for tillia honey, which increased by 13% and the lowest variation of 4% for rapeseed honey. The acidity of the treated samples decreased. The most significant decrease of 12% was found in the case of rapeseed honey, the acidity of grassland honey remained constant, and for the other types of honey, the acidity decreased by 1–2%.

Similar to the results of our study, Lazarević et al. [63] reported the lowest mean free acid value of 11.6 meq/kg for acacia honey and the highest value was 27.2 meq/kg for a sunflower honey sample. Baloś et al. [64] reported mean acidity values such as 13.08 ± 8.41 meq/kg for meadow honey, 5.44 ± 1.56 meq/kg for acacia honey, 12.17 ± 6.73 meq/kg for linden honey, 13.61 ± 6.12 meq/kg for polyfloral honey and 19.33 ± 8.70 meq/kg for forest honey. Ratiu et al. [65] analyzed from a physicochemical viewpoint several samples of honey harvested from different states of the world and reported free acidity values such as 20 meq/kg for acacia honey and 36.6 meq/kg for rapeseed honey, both harvested from Romania, 44 meq/kg for Brazilian polyfloral honey and honey samples, 80 meq/kg for polyfloral honey, 16.5 meq/kg for acacia honey, 21 meq/kg for raspberry honey, 24 meq/kg and 12.8 for rapeseed honey and 31 meq/kg for honeydew honey, all samples originating from Poland. In another study on honey, after 18 months of storage at room temperature, the acidity of the rapeseed honey samples remained constant at 22.21 ± 4.62 meq/kg, a result reported by Kedzierska et al. [66]. Janghu et al. [23] obtained similar results regarding the decrease of the acidity value of the control sample of different types of honey analyzed and the acidity of the ultrasound-treated samples. This treatment presents an advantage in terms of product stability: a lower value of honey acidity means better product stability and inhibition of microbial growth. Similarly, an increase in acidity was observed in the case of storing alfalfa and lotus honey for one year, the lowest value of acidity being 20.7 ± 2.3 meq/kg in lotus honey and the highest being 25.6 ± 2.7 meq/kg in thyme honey [67].

### 3.4. Diastase Activity 

The diastase enzyme is found naturally in honey [68]. The diastatic activity of honey is correlated with the source of nectar and with the geographical origin of honey [59,69]. Diastase activity is an indicator of honey freshness [70]. This enzyme is sensitive to temperature; its determination is important because it can indicate honey overheating but also poor storage conditions [71]. The diastase activity (DN) can be an indicator of long-term storage; its value decreases in time and also at heat treatments applied over 60 °C [72]. Results were expressed in Göthe units per gram of honey [31].

The highest values of diastase activity of the analyzed control samples were found in raspberry honey and grassland, 29.9° Göthe, and 29.7° Göthe, respectively, and the lowest values were found in the treated samples, acacia honey, rapeseed honey and honeydew honey, equal to 16.5° Göthe, 16.5° Göthe and 16.0° Göthe, respectively. Control samples from the seven types of honey analyzed had insignificant variations in diastatic activity. This parameter decreased for all samples. Within control samples, rapeseed honey had the largest variation in diastatic activity during the 9 months of our research, equal to 0.5° Göthe from 18.5° to 18.0° Göthe, and the diastatic activity of grassland honey remained constant at −29.7° Göthe.

In the case of ultrasound-treated samples, this parameter had a much broader variation than the untreated ones. In acacia honey, in the treated sample, the diastatic activity varied between 18.1° and 16.5° Göthe, for raspberry honey it varied between 27.8° and 27.1° Göthe, in tillia honey between 22.9° and 21.8° Göthe, in polyfloral honey between 17.8° and 16.3° Göthe, in rapeseed honey between 17.9° and 16.5° Göthe, in honeydew honey between 17.5° and 16.0° Göthe and in grassland honey between 29.0° and 24.4° Göthe.

These results show that the ultrasound treatment application, at a frequency of 42 kHz, has the same effect for all the types of honey subject to our analysis; specifically it acts on diastase, reducing the value of its activity in time. The decrease of diastatic activity in the treated samples is much higher than in the control samples. 

The decrease of enzyme activity in honey samples was caused by ultrasonic energy that produces cavitation and acoustic flux [73]. The latter has generated mechanical, thermal and chemical effects that have inactivated the enzymes from honey [74]. Simultaneously, the decrease in diastatic activity after ultrasound treatment was caused by the loss of enzyme biological activity as ultrasound modified the secondary and tertiary structure of the enzymes [75].

Onur et al. [20] reported different results to those obtained in our study and showed that ultrasonic treatment at a frequency of 24 kHz on sunflower, cotton and canola honey has no effect on the diastatic activity of the treated honey, this parameter remaining constant for the samples of sunflower, canola and cotton honey, equal to 13.9. Patrignani et al. [76] reported values of diastatic activity between 21.13 and 29.58 for four types of Argentinian honey. The decrease in diastatic activity was reported in a study by Kedzierska et al. [66]; during eighteen months, this quality parameter decreased by 24.4% from 21.44, results that were in accordance with our result.

### 3.5. Hydroxymethylfurfural Determination

Hydroxymethylfurfural (HMF) is a byproduct that is formed during the Maillard reaction or the decomposition of hexose in an acidic medium [77]. To these reactions are added nonenzymatic chemical changes that also produce a series of browning pigments [78]. Hydroxymethylfurfural is a cytotoxic, genotoxic and organotoxic compound [79]. Recent studies have concluded that HMF can be present in low concentrations in natural honey, being an indicator of its freshness. The concentration of HMF depends on other parameters of honey, such as light exposure, pH, acidity, water content [68,80]. According to the European legislation and the Codex Alimentarius, the concentration of HMF allowed in honey can vary up to 40 mg/kg (80 mg/kg in tropical honey) [1,38].

The temperature in ultrasonic bath increased up to 25 °C during ultrasound treatment. This temperature rise of 5 °C, from 20 °C to 25 °C, did not cause changes in the HMF content. The HMF content in the ultrasound-treated samples increased immediately after the application of the treatment and continued to increase along the 9 months of study. The increase in the HMF content in the control samples was much lower compared to the treated samples. In the case of the control samples, the smallest variation in HMF concentration was 5% in raspberry honey, and the most significant variation was 30% in honeydew honey. In the case of the treated samples, the largest variation of this parameter was found in tillia honey with 127%, and the smallest variation of 26% was in rapeseed honey. Ultrasound forms free radicals in the treated honey samples. Therefore, the stronger the ultrasound treatment, the more free radicals form, which in turn can lead to the formation of HMF [23,81,82].

Similar results to the results obtained in our study regarding the increase of HMF concentration in ultrasound-treated samples were obtained by Phawatwiangnak et al. [22] and Onur et al. [20]. Solis Silva et al. [25] showed that the largest increase in HMF content appeared after 15 min of ultrasound treatment and after 120 days of storage. Onur et al. [20] showed through their study that the ultrasonic treatment applied on three types of honey determined the increase of HMF content in canola honey from 0.90 to 1.05 ppm, in cotton honey from 1.05 to 1.15 ppm and the decrease of HMF content in sunflower honey from 2.37 to 1.57 ppm. Patrignani et al. [76] analyzed four types of honey and reported values between 3.27 mg/kg for Patagonian Forest honey and 24.7 mg/kg for Parana Delta and Islands. Isla et al. [83] reported mean HMF values between 4.0 ± 1.3 mg/kg and 49.0 ± 2.2 mg/kg for thirteen samples of Argentinian honey. The study by Kedzierska et al. [78] showed that the storage of rapeseed honey at a temperature of 20–26 °C determined an increase in the initial value of the HMF concentration of 19.74 mg/kg by 543%.

### 3.6. Color Analysis

The color of honey depends on the content of minerals, phenolic compounds, flavonoids and storage conditions [84].

**Figure 1:**
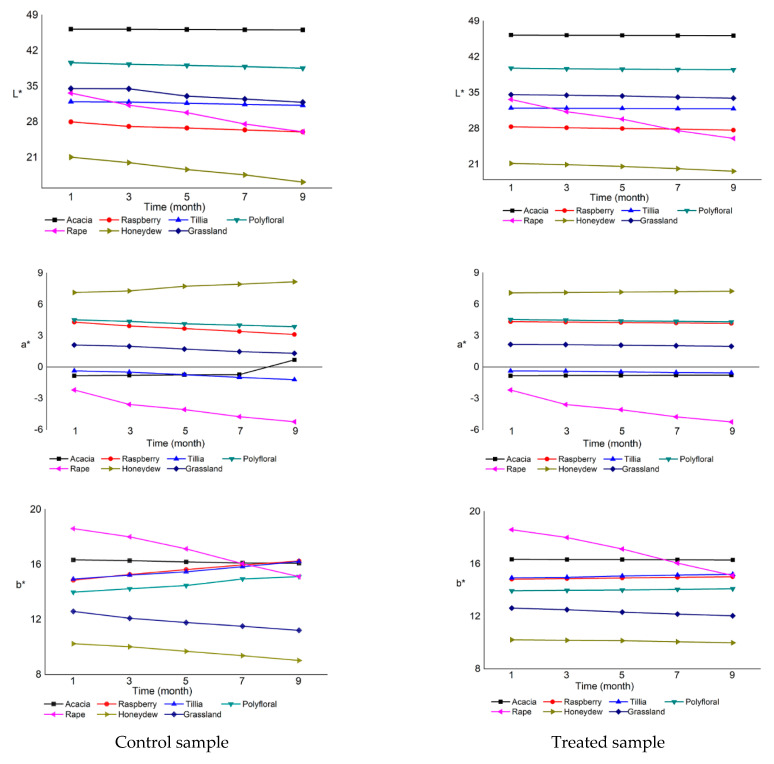
Color parameters for the seven honey samples.

The color parameter analyzed was L*, a*, b* and calculated the Chroma, Hue angle and Yellow index. These results are illustrated in Figure 1, L*, a*, b*. Modifications were more visible in the case of control samples than in those treated with ultrasound.

In the case of acacia honey, the brightness varied the least, by 0.3% for the control sample and by 0.2% for the treated sample. The brightness of other types of honey varied more. The rapeseed honey had the largest variation in the control sample, of 22%, and in the treated sample the variation was only 13%. The lowest lightness was found in the control sample of honeydew (16.17), and the highest in acacia honey (46.26). a* parameter represents the green component (negative a* values) and it was present in all samples of acacia honey, tillia honey and rapeseed honey. The highest value of the green compound was found in the ninth month, in the control sample of rapeseed honey (−5.24). The red parameter (positive values of a*) was found in raspberry honey, polyfloral honey, honeydew honey and grassland honey. The highest value of the red parameter was found in the ninth month, in the control sample of honeydew (8.17), and the lowest value was found in the control sample of grassland honey (1.32), also in the ninth month.

The brightness of the control sample of the seven types of honey varied according to the type of honey, and whether the samples were ultrasound-treated or not analyzed also varied, but it also depended on the application of ultrasound treatment or not. The crystallization tendency in acacia honey was low, having the G/F ratio = 0.62, G/W = 1.55, the brightness value was high, and rapeseed honey had a high crystallization tendency and low brightness, G/F ratio = 1.96, G/W = 2.84. The application of the ultrasonic treatment affected the later installation of the crystallization in the seven types of honey and it improved the stability of their color parameters for a longer time. The most significant variations of the color parameters were observed in rape honey; in the control sample parameter, a* varied by 139%, parameter b* by 18%, Chroma by 33% and Hue angle by 16%, and the Yellow index had the highest increase of 17% in the control sample of raspberry honey. All these variations were due to the formation of glucose crystals that lighten the colors and alter the transparency of the samples.

Regarding the Chroma parameter (Table S1), the sample values were variable; the untreated samples had a much larger variation than the treated ones. The only exception was the acacia honey with insignificant color variation because both the control sample and the ultrasound-treated one did not crystallize. The highest value of Chroma was found in rapeseed honey (6.07) and the lowest in honeydew honey (1.53). The largest variation was found in rapeseed honey (6.04–3.99) and the slightest in acacia honey (4.66–4.63).

The Hue angle had negative values in acacia, tillia and rapeseed honey, which also had negative values of the parameter a*. The largest variation was recorded in rapeseed honey and the smallest in acacia honey. The Yellow index had been more obviously modified in time for the control samples than for the ultrasound-treated samples. The lowest value of the Yellow index was found in a treated sample of acacia honey. The color of the honey samples was characterized by shades of red, yellow and green, the samples of raspberry, polyfloral, honeydew and grassland honey being located in the first quadrant of the CIE color space a* b* because the coordinates a* and b* had positive values. The negative values of the parameter a* of the honey samples of acacia, tillia, and rapeseed placed them in the second quadrant, being also characterized by shades of yellow and green.

All samples had light color due to the crystallization of glucose, which is reflected in the value of a* and b*, through a decrease in luminosity and yellow index. The lightening and the decrease of the Hue angle were observed during storage at all temperature conditions studied by Zábrodská et al. [85].

Bertoncelj et al. [86] analyzed samples from six varieties of honey from Slovenia and reported average color parameter values for a polyfloral honey such as L* = 53.87, a* = 2.25 and b* = 46.45. There were significant differences between the values of color parameters obtained in this study and a study by Halagarda et al. [87] in which twenty-two samples of eleven varieties of Polish honey were analyzed. As an exemplification, L* varied between 23.18 in honeydew honey and 59.91 in a sample of polyfloral honey, a* had values between −1.61 and 11.6 for two polyfloral honey samples, and the values of parameter b* were between 7.59 for a rapeseed honey sample and 31.33 for a polyfloral honey sample. Kuś et al. [88] reported values of the parameter L* between 41.5 in buckwheat honey and 86.2 in black locust honey, of a* between −1.6 and 31.9, and of b* between 19.6 and 69.2 in black locust and buckwheat honey, respectively. Janghu et al. [23] reported a decrease in brightness 29.1 ± 0.9 to 28.8 ± 0.3 in the ultrasound-treated sample and an increase in red (+a*) and yellow (+b*) values in the ultrasound-treated samples compared to the control samples, with values ranging from 2.21 ± 0.04 to 2.5 ± 0.3, respectively, from 18.5 ± 0.6 to 20.1 ± 0.5. Similar results were reported for floral honey in Thailand, where the smallest changes in a* and b* values were observed in ultrasound samples treated at 80 and 40% amplitude for 30 min [17].

### 3.7. Texture Analysis

**Figure 2:**
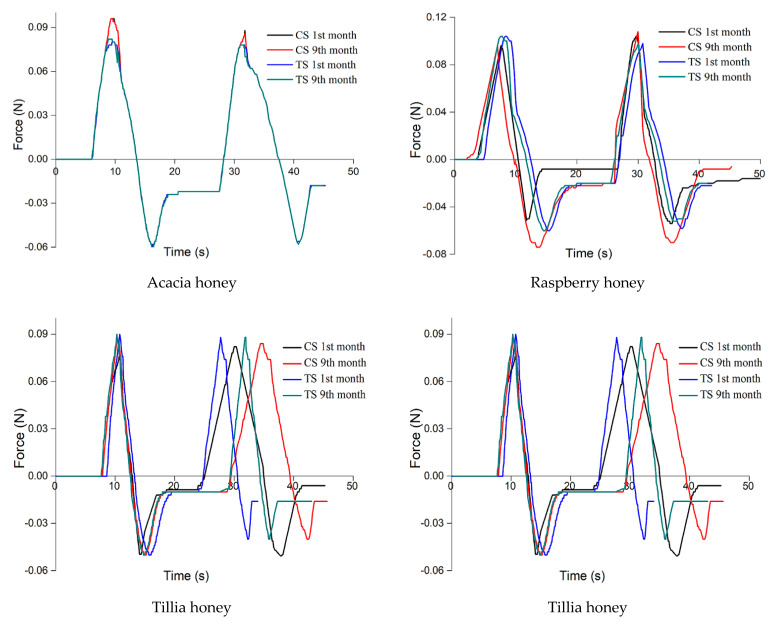

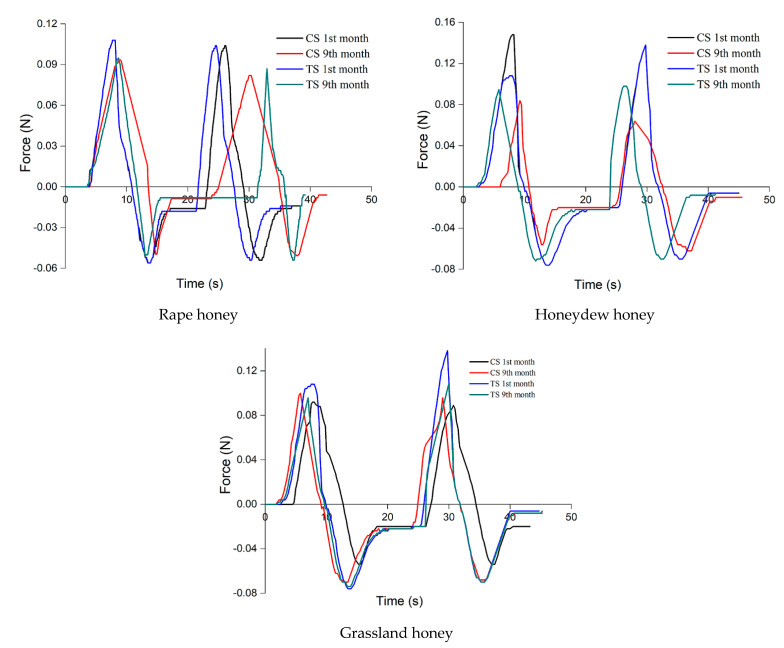
Texture analysis for the seven honey samples (CS: control samples, TS: treated samples).

Texture analysis (Figure 2) showed that the ultrasound-treated samples kept the parameters unchanged for a longer time or changed less because the applied treatment decreased the crystal formation rate, while the control samples changed the parameter texture in the first months of research.

The texture parameters of the acacia honey sample varied the least, due to its much lower glucose content, resulting in the formation of a small number of crystals, a hypothesis supported by the analysis of crystals. The hardness increased by 2%, springiness by 3%, cohesiveness and adhesiveness increased by 1%, viscosity by 5%, chewiness by 7% and gumminess by 4%.

The largest variation of the texture parameters was observed in rapeseed honey.

The texture parameters of the untreated rapeseed honey sample varied the most, hardness and gumminess varying by 237% and 239%, respectively, viscosity by 126%, cohesiveness by 21%, adhesiveness by 57% and chewiness by 64%. This correlated with the changes in color, the significant decrease in sample brightness and the massive presence of glucose crystals. The increase in hardness, viscosity, cohesiveness, gumminess and chewiness, as well as the decrease in elasticity and adhesiveness is explained by the change in the interatomic and intermolecular forces caused by the formation of glucose crystals in the supersaturated solution.

The results showed that as the crystal phase increased due to the crystallization of glucose in the control samples, a solid structure was formed in which the crystals created a matrix characterized by a cohesiveness that increased in time, thus changing the texture of the honey samples. Once the crystallization process started, the hardness, viscosity, chewiness and cohesiveness increased due to the increasing number and size of crystals. The adhesiveness and springiness decreased in all samples, and the gumminess varied differently depending on the botanical origin of the honey.

One of the findings of our study regarding the increase of honey viscosity due to the crystallization process and the possibility to decrease it by applying low- and medium-intensity and low-frequency ultrasonic treatment are consistent with the works of other scholars on this matter. Ultrasonic treatment can be a viable method to alter glucose crystallization and to prevent the formation of crystallized honey [89].

Recrystallization appeared in ultrasound-treated polyfloral honey samples after 100 days, with a hardness value of 100 N, and in ultrasound-treated buckwheat honey samples after 150 days, with a hardness value of 140 N. The hardness of the ultrasound-treated samples varied during storage under the influence of the variables of the treatment itself (frequency, duration, amplitude). The hardness of polyfloral honey had the lowest value and recrystallized the fastest, the hardness value of lime honey was slightly higher, and buckwheat honey had the highest hardness value and recrystallized the slowest [89].

Onur et al. [20] reported in their study that the viscosity of ultrasound-treated samples was 10.99 ± 2.11 Pa·s and was approximately ten times lower than the viscosity of untreated samples, which were equal to 100.72 ± 4.47 Pa·s. Kabbani et al. [90] studied the viscosity of rosemary honey during ultrasound treatment and reported a decrease in this physical parameter of approximately ten-fold from 12,733 ± 25 mPa·s to 1266 ± 11 mPa·s after 9 min of ultrasound treatment.

### 3.8. Microscopic Crystals Analysis

**Figure 3:**
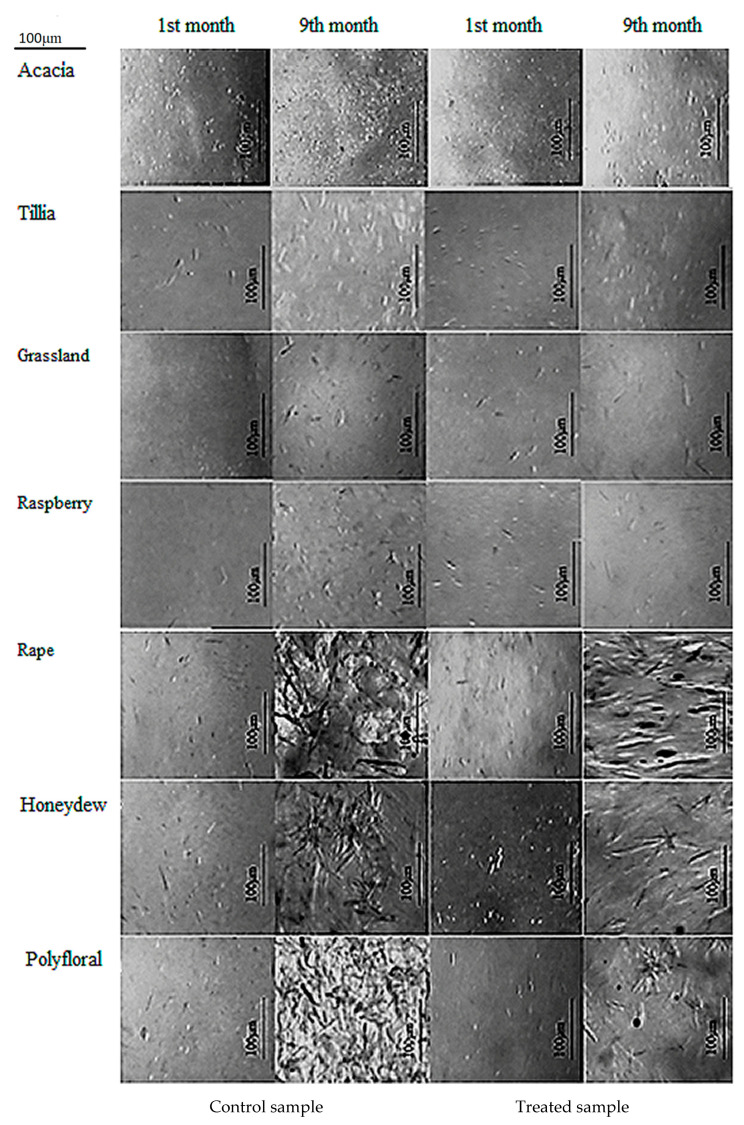
Microscopic images of the crystals of the seven types of honey analyzed.

Figure 3 shows the differences between the crystals of the seven types of honey analyzed in the first month and last month.

From the presented images, it is observed in all samples, both treated and untreated, in the first month there are a few, fine, uniform, small dimensions crystals. The microscopic images from the last month showed differences between the types of honey analyzed and between the treated and control samples of the same honey. In acacia honey, in the first month, fine crystals were observed, small and distributed relatively evenly. In the last month, the size of the crystals did not change considerably, but they were more numerous in the control sample than in the one treated with ultrasound. Microscopic images for tillia honey showed in the first month, both in the control and in the ultrasound-treated samples, elongated crystals, unevenly distributed, and their frequency in the field was lower in the treated sample than in the control sample. In the ninth month, the number, size and shape of the crystals changed in the control sample.

Few crystals were present in grassland and raspberry honey in the first month, and in the ninth month they were presented in the form of crystal bouquets, uniform agglomerations of small crystals in the control sample and less frequently in the treated sample, the crystals being larger for raspberry honey. In grassland honey, in the last month, the images showed larger crystals, shaped like needles; their frequency in the field was lower compared to the large number and smaller size of crystals in the control sample.

In the first month, the polyfloral honey presented in both treated and untreated sample a few small dimensions crystals, with predominantly elongated shapes, along with other shapes. In the ninth month, the control sample presented an agglomeration of larger size crystals, and the ultrasound-treated sample showed bouquets of needle-shaped crystals with a much lower frequency in the field than the control sample.

For honeydew honey, significant differences were observed in the ninth month microscopic images; respectively, the crystal bouquets were observed in both the treated and untreated samples. In the control sample, the crystals were larger and more numerous, and in the treated sample, ultrasonic crystals in the form of a bouquet of needles were smaller and fewer. Rapeseed honey did not present any special crystals in the first month, both in the treated and the untreated sample. Yet, in the ninth month, large pentagonal and hexagonal crystals were observed, covering the entire field. Meanwhile, in the ultrasound-treated sample a reduction in both frequency and size of the crystals was observed.

Acacia honey best preserved its crystalline properties in time and rapeseed honey underwent the most radical changes. These changes were correlated with the glucose and fructose content of the analyzed samples, the glucose being the one that crystallizes faster. Furthermore, the color and texture parameters changed, once the crystals appeared. As crystals appeared, the brightness of the samples decreased, the values of hardness, cohesiveness, viscosity and gumminess increased, and springiness, adhesiveness and chewiness decreased.

Kabbani et al. [90] studied the behavior of rosemary honey crystals during ultrasound treatment and reported the liquefaction of samples being an effective way to reduce the size of the glucose crystals while also improving the uniform distribution in the product.

### 3.9. Microbiological Analysis

The values obtained for parameters of yeasts, molds, *Bacillus cereus*, total coliforms (TC) and SPC for control samples and ultrasound-treated samples were within acceptable limits according to the latest standards.

Honey samples analyzed presented no health hazards, and the pathogenic microflora was undetectable. The quality of the samples was superior from a microbiological viewpoint, in correlation with the values of the parameters presented in Table 2.

The application of ultrasound treatment reduced or maintained the values of the microbiological parameters. *Bacillus cereus* and total coliforms were undetectable in all honey samples analyzed.

In the ultrasound-treated samples of acacia honey, honeydew honey and grassland honey, yeasts, molds or SPCs were undetectable. For control samples, SPC values were <10–10 cfu/g in acacia honey, 30–40 cfu/g in raspberry honey, 20–30 cfu/g in tillia honey, <10–20 cfu/g in polyfloral honey, 40–50 cfu/g in rapeseed honey and <10 cfu/g in honeydew and grassland honey. Ultrasound-treated samples maintained their SPC parameter levels or were thus reduced: <10–10 cfu/g in raspberry honey and tillia honey, <10 cfu/g in polyfloral honey and 20 cfu/g in rapeseed honey. Yeasts were undetectable in ultrasound samples of acacia, tillia, polyfloral, rapeseed, honeydew and grassland honey, and raspberry honey had a value of <10–10 cfu/g. The yeasts were present in the control samples, the values varying thus: <10–10 cfu/g in acacia honey, 20–30 cfu/g in raspberry honey, 10–30 cfu/g in tillia honey, <10–40 cfu/g in polyfloral honey, 20–40 cfu/g in rapeseed honey, <10–20 cfu/g in honeydew and grassland honey. In the acacia honey, in both the control sample and the treated one, molds were not detected. Furthermore, the treated samples of honeydew and grassland honey did not show molds. In the control sample of honeydew honey, mold was detected: <10–10 cfu/g, respectively, <10 cfu/g in grassland honey, <10–30 cfu/g in raspberry honey, 10–40 cfu/g in tillia honey, 20–40 cfu/g in polyfloral honey, <10–20 cfu/g in rapeseed honey. In the ultrasound-treated samples, mold values varied as follows: <10 cfu/g in raspberry honey and in rapeseed honey, 10 cfu/g in tillia and polyfloral honey. 

The reduction and inactivation of microorganisms in the analyzed honey samples are the result of physicochemical processes that take place during treatment. Physical processes are based on rapidly changing mechanical loads, cavitation with several related phenomena such as cellular resonance. Chemical processes involve the generation of free radicals through the decomposition of water into oscillating bubbles. The cell membranes of microorganisms are perforated with the formation of free radicals, and extrusion of the intracellular matrix destroys the microorganisms [91,92,93,94,95].

Similar results were obtained by Onur et al. [20], Chaven et al. [96] and Yikmiş et al. [91] on reducing the values of microbiological parameters. Onur et al. reported a decrease of total coliforms from (5.2 ± 0.2) 10^3^ to (1.8 ± 0.1) 10^2^ or removing yeasts from (0.2 ± 0.1) 10^2^ and molds from (0.6 ± 0.1) 10^5^. Similar results to those obtained by us were obtained by Starek et al. [92], who reported improvement in the microbiological quality of tomato juice by microbial reduction after ultrasound treatment (at 40 W for five minutes and at 28 W for ten minutes).

### 3.10. Statistical Analysis

The statistical analysis showed that there are strong correlations between analyzed parameters. All values are significant at *p* < 0.05. Pearson correlation (Supplementary Material Table S3) was performed for each type of analyzed untreated honey sample. The Table S3a (acacia honey) shows that there are positive correlations between the G/F ratio and the G/W ratio (0.961), HMF (0.945), between the G/W ratio and HMF (0.950), between color parameter b *, Chroma and springiness (0.945), adhesiveness (0.915) and negative correlations between G/F ratio and diastase activity (ID), (−0.960), water activity (−0.940), between G/W ratio and ID (−0.976), water activity (−0869). Both positive and negative correlations were identified between color and texture parameters. As the brightness decreases, hardness, cohesiveness, viscosity, chewiness and gumminess increase, which indicates the installation of the crystallization process. Table S3b (raspberry honey) presents a positive correlation of glucose content with HMF and water activity (1.000–perfect correlation), hardness (0.905), between G/F ratio and the ID (0.881), and negative correlation between diastase activity (ID) and water content, fructose and sucrose. Between the color and texture parameters, there were strongly positive and negative correlations. The brightness decreased with an increase in viscosity. In Table S3c (tillia honey) positive correlation was observed between fructose content and adhesiveness (0.871) and negative correlation with hardness (−0.912). Both positive and negative correlations existed between color and some texture parameters such as viscosity, chewiness and gumminess. Positive correlations exist in Table S3d (polyfloral honey) between glucose content and sucrose (0.954), HMF (0.877), adhesiveness (0.992) and negative correlation with ID (−0.944). Here, the HMF content increases proportionally to glucose content and decreases proportionally to the value of the diastase (ID) activity. Water activity showed positive correlations with the parameter L*(0.851), springiness (0.900), adhesiveness (0.859), chewiness (0.887) and gumminess (0.916) and negative correlation with viscosity (−0.891). The color parameters correlated positively and negatively with some texture parameters such as hardness, springiness, cohesiveness, viscosity, chewiness and gumminess. Table S3e with the correlations obtained for the analyzed samples of rapeseed honey show negative correlations between water content and HMF (−0.932), cohesiveness (−0.953), fructose content and HMF (−0.977), cohesiveness (−0.940), between diastatic activity and gumminess (−0.884). Positive correlations were observed between glucose content and water activity (1.000), G/F and G/W ratios correlated with a texture-cohesiveness parameter (0.933 and 0.953). The analyzed color parameters correlated positively and negatively with some texture parameters such as hardness, springiness, cohesiveness and adhesiveness. Table S3f (honeydew honey) shows strong negative correlations between glucose content and HMF (−0.897), diastase activity and HMF (−0.932), between sucrose and viscosity (−0.872). HMF was positively correlated with springiness (0.864) and fructose (0.897). Color parameters correlated with hardness, cohesiveness, adhesiveness, viscosity, chewiness and gumminess. The brightness decreased along with decreasing adhesiveness and gumminess. Table S3g, containing the correlations obtained for the analyzed samples of grassland honey, show positive correlations between fructose and HMF (0.892), adhesiveness (0.875), gumminess (0.912), between sucrose content and water activity (1.000). Negative correlations were observed between G/F ratio and adhesiveness (−0.888) and gumminess (−0.954). Analyzed color parameters correlated positively and negatively with some texture parameters such as hardness and viscosity. Springiness was correlated only with parameters L*, a* and H.

**Figure 4:**
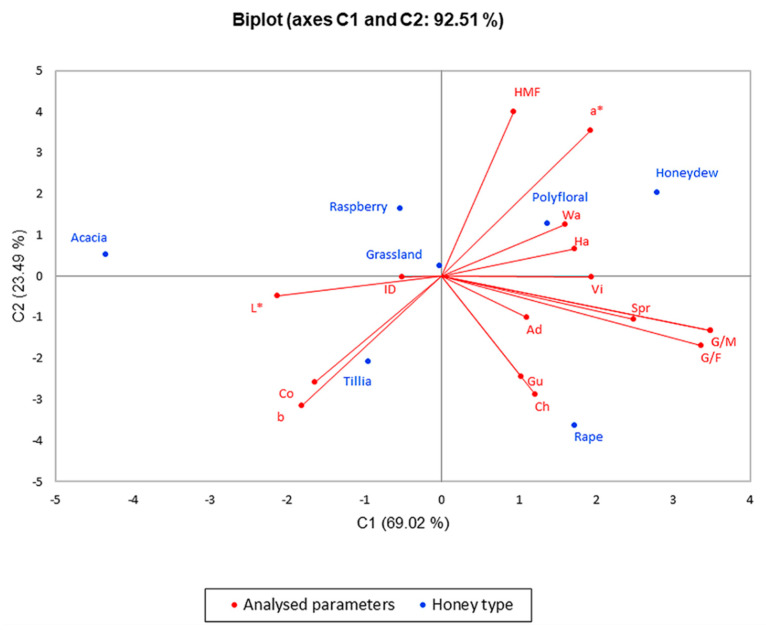
Principal component analysis (PCA) regarding the honey types in the first month of analyses.

**Figure 5:**
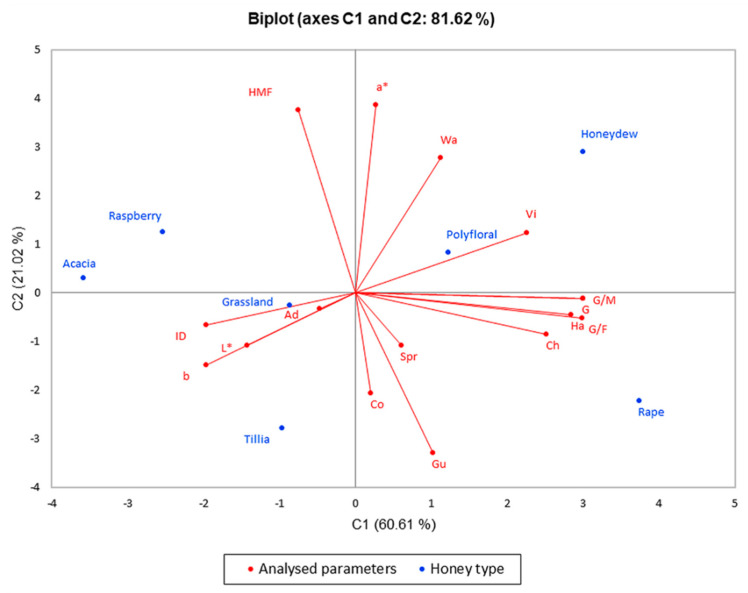
Principal component analysis regarding the honey types in the last month of analyses.

To emphasize the difference between the seven types of honey, and the parameters analyzed in the first and last month, we performed the analysis of the main components (PCA), and the results are presented in the biplot in Figure 4 and Figure 5. The PCA method limited all data in two main components, the active variables being glucose content (G), G/F ratio, G/M ratio, diastase index (ID), hydroxymethylfurfural concentration (HMF), water activity (Wa), some color parameters, and texture parameters as hardness (Ha), springiness (Spr), adhesiveness (Ad), cohesiveness (Co), viscosity (Vi), chewiness (Ch) and gumminess (Gu). The significance level alpha was 0.05.

The PCA method narrowed the data into two main components covering 92.51% (for the results obtained in the first month of the research) and 81.62% (for the results obtained in the last month of the research) of the variability; thus, PC1 represented 69.02% and 60.61% of data, respectively, and PC2 represented 23.49% and 21.02%, respectively. 

From PCA illustrated in Figure 4, one can observe that polyfloral honey, honeydew honey, rapeseed, tillia and grassland have similarities in results, and they have higher values of water activity, hardness, viscosity, adhesiveness, gumminess, cohesiveness, chewiness and G/F, G/W ratio, diastase activity, HMF concentration than acacia honey.

Figure 5 shows the distribution of physicochemical parameters changes, texture and color parameter changes and, to conclude, the application of ultrasound treatment is significantly correlated with glucose content, G/F and G/W ratios and hardness with higher values in the case of rapeseed honey, which crystallizes rapidly. On the opposite side of the graph, there are acacia and raspberry honey that had lower values of the parameters listed above, an indication of a slower crystallization compared to rapeseed honey. These two figures illustrating PCA show a significant difference in these seven types of honey, a difference that consists in high glucose content in rapeseed honey and its influence on crystallization, which is confirmed by the texture of the samples analyzed.

Ultrasonic treatment has improved the microbiological quality of the analyzed honey. Yeasts were found in 100% of the untreated samples. Yeasts were present in 14% of the ultrasound-treated samples. Molds were found in 85% of the control samples. The applied ultrasound treatment reduced the number of molds by 66%. Molds were found in 57% of the treated samples. The SPC results show that for 100% of the untreated samples this parameter was detectable. SPC was reduced by ultrasonication in 42% of the treated samples.

## 4. Conclusions

Honey crystallization causes changes in color, textural and rheological parameters, phase separation. It affects consumer acceptance and creates logistic difficulties for producers in the process of storage and handling of honey. There are serious concerns, on these grounds, towards preventing this process.

Significant changes in physicochemical, textural and microbiological parameters were found in the control samples. Ultrasound-treated samples kept their properties longer and the parameter changes were minor. Sugar content remained constant for both the treated and control samples.

Microbiological parameters have been improved by ultrasound treatment.

Statistical analysis showed strong correlations of the G/F and G/W ratios with some texture and color parameters that characterize the crystallization process.

This research highlights the effect of applying ultrasonic treatment to different types of honey on physicochemical parameters, color, texture parameters, microbiological parameters and the appearance of crystals in time. The analyses carried out made it possible to highlight the importance of treatment to prevent microbial growth in all honey types analyzed. Furthermore, the effect of ultrasound treatment on quality parameters analyzed was different for each type of honey and this was also highlighted. The positive influence of ultrasound on the texture of honey, the maintenance of it for a longer period of time (nine months) was evidenced by texture curves and microbiological results. The ultrasound treatment applied did not increase the HMF concentration above 40 mg/kg (maximum limit according to EU legislation).

## Figures and Tables

**Table 1:** Physicochemical analysis of honey samples.

Honey Variety		Month	Water Content, %	Glucose, g/100 g	Fructose, g/100 g	Sucrose, g/100 g	G/F	G/W	Acidity, mEq/kg	Diastase Activity, °Göthe	HMF, mg/kg	Water Activity
Acacia	Control sample	1st	17.50 ± 0.46	27.24 ± 0.15	43.58 ± 0.17	0.90 ± 0.17	0.62 ± 0.32	1.55 ± 0.72	13.5 ± 0.47	18.3 ± 0.38	0.59 ± 0.19	0.56 ± 0.08
		3rd	17.50 ± 0.14	27.24 ± 0.45	43.56 ± 0.82	0.89 ± 0.21	0.62 ± 0.07	1.55 ± 0.56	13.5 ± 0.53	18.3 ± 0.20	0.61 ± 0.32	0.56 ± 0.10
		5th	17.50 ± 0.61	27.24 ± 0.12	43.57 ± 0.34	0.89 ± 0.09	0.62 ± 0.15	1.55 ± 0.38	13.5 ± 0.32	18.3 ± 0.57	0.62 ± 0.62	0.56 ± 0.16
		7th	17.60 ± 0.25	27.24 ± 0.08	43.56 ± 0.48	0.89 ± 0.16	0.62 ± 0.12	1.54 ± 0.02	14.5 ± 0.19	18.3 ± 0.42	0.64 ± 0.45	0.56 ± 0.26
		9th	17.60 ± 0.02	27.34 ± 0.76	43.56 ± 0.19	0.90 ± 0.04	0.62 ± 0.09	1.55 ± 0.09	14.6 ± 0.14	18.2 ± 0.24	0.65 ± 0.09	0.56 ± 0.32
	Ultrasound treated sample	1st	17.50 ± 0.23	27.24 ± 0.57	43.58 ± 0.21	0.89 ± 0.16	0.62 ± 0.25	1.55 ± 0.17	13.9 ± 0.29	18.1 ± 0.07	0.68 ± 0.04	0.55 ± 0.38
		3rd	17.30 ± 0.47	27.25 ± 0.36	43.64 ± 0.04	0.89 ± 0.04	0.62 ± 0.12	1.57 ± 0.28	13.7 ± 0.09	17.1 ± 0.11	1.04 ± 0.30	0.55 ± 0.12
		5th	17.30 ± 0.19	27.25 ± 0.01	43.64 ± 0.01	0.89 ± 0.64	0.62 ± 0.09	1.57 ± 0.01	13.8 ± 0.25	17.0 ± 0.23	1.18 ± 0.47	0.55 ± 0.25
		7th	17.20 ± 0.21	27.25 ± 0.17	43.64 ± 0.47	0.89 ± 0.25	0.62 ± 0.53	1.58 ± 0.29	13.7 ± 0.47	16.8 ± 0.25	1.35 ± 0.23	0.55 ± 0.06
		9th	17.20 ± 0.32	27.25 ± 0.04	43.64 ± 0.19	0.89 ± 0.38	0.62 ± 0.33	1.58 ± 0.11	13.7 ± 0.07	16.5 ± 0.12	1.45 ± 0.07	0.55 ± 0.47
Raspberry	Control sample	1st	16.10 ± 0.13	29.50 ± 0.64	38.52 ± 0.14	2.09 ± 0.12	0.76 ± 0.20	1.83 ± 0.43	13.0 ± 0.64	29.9 ± 0.09	0.78 ± 0.12	0.62 ± 0.21
		3rd	16.10 ± 0.42	29.50 ± 0.23	38.52 ± 0.09	2.09 ± 0.06	0.76 ± 0.19	1.83 ± 0.01	13.0 ± 0.38	29.9 ± 0.32	0.78 ± 0.01	0.62 ± 0.23
		5th	16.10 ± 0.01	29.50 ± 0.14	38.52 ± 0.23	2.09 ± 0.07	0.76 ± 0.45	1.83 ± 0.30	13.0 ± 0.09	29.9 ± 0.25	0.78 ± 0.07	0.62 ± 0.11
		7th	16.20 ± 0.47	29.50 ± 0.09	38.52 ± 0.41	2.09 ± 0.47	0.76 ± 0.06	1.82 ± 0.21	13.5 ± 0.14	29.9 ± 0.30	0.79 ± 0.09	0.62 ± 0.04
		9th	16.20 ± 0.10	29.50 ± 0.21	38.52 ± 0.57	2.09 ± 0.32	0.76 ± 0.07	1.82 ± 0.53	13.7 ± 0.11	29.8 ± 0.21	0.82 ± 0.12	0.62 ± 0.42
	Ultrasound treated sample	1st	16.20 ± 0.47	29.50 ± 0.19	38.53 ± 0.13	2.10 ± 0.12	0.76 ± 0.29	1.82 ± 0.12	13.1 ± 0.63	27.8 ± 0.04	0.81 ± 0.30	0.62 ± 0.12
		3rd	16.50 ± 0.09	29.50 ± 0.04	38.53 ± 0.02	2.10 ± 0.47	0.76 ± 0.30	1.78 ± 0.07	13.0 ± 0.32	27.9 ± 0.46	1.10 ± 0.21	0.62 ± 0.06
		5th	16.50 ± 0.53	29.50 ± 0.01	38.53 ± 0.09	2.10 ± 0.46	0.76 ± 0.07	1.77 ± 0.42	13.0 ± 0.01	27.6 ± 0.09	1.19 ± 0.47	0.62 ± 0.53
		7th	16.50 ± 0.64	29.50 ± 0.22	38.54 ± 0.19	2.10 ± 0.21	0.76 ± 0.32	1.78 ± 0.09	13.0 ± 0.64	27.5 ± 0.23	1.27 ± 0.19	0.62 ± 0.07
		9th	16.50 ± 0.15	29.50 ± 0.01	38.54 ± 0.75	2.10 ± 0.09	0.76 ± 0.64	1.78 ± 0.12	12.8 ± 0.06	27.1 ± 0.64	1.38 ± 0.63	0.62 ± 0.12
Tillia	Control sample	1st	17.20 ± 0.15	35.36 ± 0.23	30.09 ± 0.04	1.52 ± 0.21	1.17 ± 0.09	2.05 ± 0.30	20.0 ± 0.01	23.8 ± 0.11	0.36 ± 0.12	0.52 ± 0.06
		3rd	17.10 ± 0.12	35.36 ± 0.04	30.08 ± 0.61	1.52 ± 0.19	1.17 ± 0.11	2.06 ± 0.01	20.0 ± 0.29	23.8 ± 0.32	0.36 ± 0.01	0.52 ± 0.21
		5th	17.10 ± 0.04	35.36 ± 0.01	30.08 ± 0.42	1.52 ± 0.01	1.17 ± 0.06	2.06 ± 0.06	20.0 ± 0.12	23.8 ± 0.63	0.36 ± 0.25	0.52 ± 0.23
		7th	17.10 ± 0.42	35.36 ± 0.64	30.08 ± 0.53	1.53 ± 0.80	1.17 ± 0.44	2.06 ± 0.29	22.0 ± 0.21	23.7 ± 0.01	0.39 ± 0.42	0.52 ± 0.29
		9th	17.10 ± 0.21	35.36 ± 0.24	30.08 ± 0.01	1.53 ± 0.49	1.17 ± 0.63	2.06 ± 0.30	22.6 ± 0.14	23.6 ± 0.30	0.40 ± 0.00	0.52 ± 0.01
	Ultrasound treated sample	1st	17.10 ± 0.41	35.35 ± 0.53	30.09 ± 0.04	1.53 ± 0.45	1.17 ± 0.12	2.06 ± 0.29	20.1 ± 0.42	22.9 ± 0.27	0.43 ± 0.12	0.52 ± 0.64
		3rd	17.00 ± 0.14	35.35 ± 0.08	30.09 ± 0.09	1.52 ± 0.14	1.17 ± 0.07	2.07 ± 0.25	20.0 ± 0.30	22.7 ± 0.64	0.79 ± 0.42	0.52 ± 0.01
		5th	17.00 ± 0.38	35.35 ± 0.42	30.09 ± 0.23	1.52 ± 0.07	1.17 ± 0.30	2.07 ± 0.01	20.0 ± 0.11	22.4 ± 0.29	0.85 ± 0.06	0.52 ± 0.32
		7th	17.00 ± 0.07	35.33 ± 0.01	30.09 ± 0.42	1.52 ± 0.32	1.17 ± 0.11	2.07 ± 0.63	19.8 ± 0.38	22.1 ± 0.07	0.94 ± 0.25	0.52 ± 0.06
		9th	17.00 ± 0.15	35.33 ± 0.33	30.09 ± 0.06	1.53 ± 0.09	1.17 ± 0.01	2.07 ± 0.06	19.8 ± 0.64	21.8 ± 0.01	0.98 ± 0.30	0.52 ± 0.09
Polyfloral	Control sample	1st	17.10 ± 0.09	40.16 ± 0.15	28.80 ± 0.21	1.23 ± 0.39	1.39 ± 0.07	2.34 ± 0.09	27.0 ± 0.09	19.2 ± 0.12	0.95 ± 0.14	0.58 ± 0.07
		3rd	17.20 ± 0.10	40.16 ± 0.27	28.80 ± 0.38	1.23 ± 0.36	1.39 ± 0.07	2.33 ± 0.53	27.0 ± 0.29	19.2 ± 0.63	0.96 ± 0.38	0.57 ± 0.21
		5th	17.10 ± 0.32	40.16 ± 0.64	28.80 ± 0.14	1.23 ± 0.21	1.39 ± 0.06	2.34 ± 0.04	27.0 ± 0.01	19.2 ± 0.32	0.97 ± 0.29	0.57 ± 0.11
		7th	17.10 ± 0.53	40.16 ± 0.12	28.80 ± 0.29	1.23 ± 0.17	1.39 ± 0.07	2.34 ± 0.12	29.2 ± 0.11	19.0 ± 0.07	0.99 ± 0.42	0.57 ± 0.29
		9th	17.10 ± 0.02	40.16 ± 0.61	28.80 ± 0.04	1.23 ± 0.08	1.39 ± 0.14	2.34 ± 0.21	29.4 ± 0.12	19.0 ± 0.29	1.01 ± 0.04	0.57 ± 0.07
	Ultrasound treated sample	1st	17.30 ± 0.21	40.17 ± 0.04	28.80 ± 0.53	1.24 ± 0.25	1.39 ± 0.30	2.32 ± 0.25	27.0 ± 0.04	17.8 ± 0.22	1.27 ± 0.11	0.58 ± 0.25
		3rd	17.10 ± 0.25	40.17 ± 0.26	28.80 ± 0.14	1.25 ± 0.21	1.39 ± 0.07	2.34 ± 0.21	26.9 ± 0.21	17.1 ± 0.53	1.25 ± 0.09	0.58 ± 0.07
		5th	17.10 ± 0.32	40.17 ± 0.35	28.80 ± 0.04	1.25 ± 0.29	1.39 ± 0.12	2.34 ± 0.06	27.0 ± 0.11	16.9 ± 0.11	1.43 ± 0.10	0.58 ± 0.32
		7th	17.10 ± 0.55	40.17 ± 0.12	28.80 ± 0.28	1.25 ± 0.06	1.39 ± 0.19	2.34 ± 0.18	27.0 ± 0.25	16.4 ± 0.63	1.61 ± 0.07	0.58 ± 0.06
		9th	17.10 ± 0.38	40.17 ± 0.64	28.80 ± 0.23	1.25 ± 0.25	1.39 ± 0.08	2.34 ± 0.07	27.0 ± 0.04	16.3 ± 0.23	1.72 ± 0.30	0.58 ± 0.25
Rapeseed	Control sample	1st	16.60 ± 0.23	46.30 ± 0.09	23.70 ± 0.21	0.97 ± 0.11	1.95 ± 0.09	2.78 ± 0.29	23.6 ± 0.25	18.5 ± 0.21	0.31 ± 0.04	0.61 ± 0.07
		3rd	16.70 ± 0.11	46.30 ± 0.37	23.70 ± 0.04	0.95 ± 0.07	1.95 ± 0.09	2.77 ± 0.32	23.6 ± 0.29	18.5 ± 0.29	0.31 ± 0.25	0.61 ± 0.25
		5th	16.70 ± 0.47	46.30 ± 0.18	23.70 ± 0.16	0.95 ± 0.29	1.95 ± 0.29	2.77 ± 0.12	23.8 ± 0.22	18.4 ± 0.63	0.33 ± 0.12	0.61 ± 0.29
		7th	16.70 ± 0.21	46.30 ± 0.67	23.70 ± 0.24	0.95 ± 0.21	1.95 ± 0.25	2.77 ± 0.22	24.3 ± 0.63	18.2 ± 0.04	0.35 ± 0.11	0.61 ± 0.06
		9th	16.70 ± 0.19	46.30 ± 0.58	23.70 ± 0.33	0.95 ± 0.01	1.95 ± 0.06	2.77 ± 0.32	24.7 ± 0.07	18.0 ± 0.25	0.35 ± 0.07	0.61 ± 0.21
	Ultrasound treated sample	1st	16.50 ± 0.64	46.30 ± 0.45	23.60 ± 0.21	0.95 ± 0.17	1.96 ± 0.07	2.80 ± 0.22	23.6 ± 0.64	17.9 ± 0.21	0.60 ± 0.29	0.61 ± 0.07
		3rd	16.50 ± 0.08	46.30 ± 0.04	23.60 ± 0.13	0.95 ± 0.41	1.96 ± 0.09	2.80 ± 0.29	23.6 ± 0.30	17.8 ± 0.12	0.71 ± 0.04	0.61 ± 0.06
		5th	16.40 ± 0.13	46.30 ± 0.41	23.60 ± 0.04	0.95 ± 0.21	1.96 ± 0.04	2.82 ± 0.19	23.6 ± 0.07	17.7 ± 0.53	0.71 ± 0.30	0.61 ± 0.25
		7th	16.30 ± 0.47	46.30 ± 0.64	23.60 ± 0.64	0.95 ± 0.47	1.96 ± 0.12	2.84 ± 0.64	23.4 ± 0.06	17.1 ± 0.09	0.74 ± 0.10	0.61 ± 0.64
		9th	16.30 ± 0.38	46.30 ± 0.14	23.60 ± 0.42	0.95 ± 0.01	1.96 ± 0.31	2.84 ± 0.07	23.4 ± 0.42	16.5 ± 0.38	0.76 ± 0.23	0.60 ± 0.12
Honeydew	Control sample	1st	16.20 ± 0.04	42.60 ± 0.38	27.70 ± 0.02	0.87 ± 0.38	1.53 ± 0.23	2.62 ± 0.29	14.0 ± 0.01	17.6 ± 0.25	0.96 ± 0.25	0.61 ± 0.07
		3rd	16.00 ± 0.12	42.60 ± 0.08	27.70 ± 0.19	0.87 ± 0.47	1.53 ± 0.30	2.66 ± 0.25	14.0 ± 0.32	17.6 ± 0.01	1.01 ± 0.06	0.62 ± 0.21
		5th	16.00 ± 0.28	42.60 ± 0.01	27.70 ± 0.44	0.87 ± 0.53	1.53 ± 0.04	2.66 ± 0.01	14.0 ± 0.48	17.6 ± 0.30	1.01 ± 0.47	0.62 ± 0.30
		7th	16.00 ± 0.32	42.60 ± 0.20	27.70 ± 0.47	0.87 ± 0.01	1.53 ± 0.12	2.66 ± 0.07	14.0 ± 0.25	17.5 ± 0.48	1.03 ± 0.29	0.62 ± 0.07
		9th	16.00 ± 0.19	42.60 ± 0.04	27.70 ± 0.01	0.87 ± 0.14	1.53 ± 0.07	2.66 ± 0.04	14.2 ± 0.41	17.4 ± 0.23	1.03 ± 0.38	0.62 ± 0.10
	Ultrasound treated sample	1st	16.00 ± 0.42	42.59 ± 0.25	27.71 ± 0.30	0.87 ± 0.21	1.53 ± 0.07	2.66 ± 0.34	14.0 ± 0.12	17.5 ± 0.07	1.25 ± 0.22	0.61 ± 0.29
		3rd	15.90 ± 0.01	42.59 ± 0.07	27.71 ± 0.48	0.87 ± 0.29	1.53 ± 0.30	2.67 ± 0.35	13.9 ± 0.21	17.1 ± 0.63	1.38 ± 0.10	0.61 ± 0.07
		5th	15.90 ± 0.54	42.59 ± 0.64	27.71 ± 0.19	0.87 ± 0.25	1.53 ± 0.29	2.67 ± 0.29	13.7 ± 0.06	17.0 ± 0.42	1.48 ± 0.38	0.61 ± 0.19
		7th	15.90 ± 0.04	42.59 ± 0.04	27.71 ± 0.01	0.87 ± 0.30	1.53 ± 0.06	2.67 ± 0.27	13.6 ± 0.30	16.2 ± 0.38	1.66 ± 0.12	0.61 ± 0.42
		9th	15.80 ± 0.12	42.59 ± 0.18	27.71 ± 0.63	0.87 ± 0.12	1.53 ± 0.14	2.69 ± 0.25	13.6 ± 0.01	16.0 ± 0.19	1.69 ± 0.14	0.61 ± 0.53
Grassland	Control sample	1st	16.10 ± 0.38	36.20 ± 0.43	38.60 ± 0.38	1.18 ± 0.07	0.93 ± 0.29	2.24 ± 0.63	17.6 ± 0.07	29.7 ± 0.53	0.55 ± 0.09	0.55 ± 0.25
		3rd	16.10 ± 0.53	36.20 ± 0.39	38.60 ± 0.21	1.16 ± 0.32	0.93 ± 0.18	2.24 ± 0.14	17.6 ± 0.09	29.7 ± 0.46	0.54 ± 0.29	0.55 ± 0.21
		5th	16.00 ± 0.64	36.20 ± 0.32	38.60 ± 0.42	1.16 ± 0.29	0.93 ± 0.12	2.26 ± 0.22	17.6 ± 0.32	29.7 ± 0.09	0.55 ± 0.30	0.55 ± 0.11
		7th	16.10 ± 0.14	36.20 ± 0.21	38.60 ± 0.01	1.16 ± 0.11	0.93 ± 0.30	2.24 ± 0.12	18.0 ± 0.47	29.7 ± 0.48	0.58 ± 0.47	0.55 ± 0.07
		9th	16.10 ± 0.18	36.20 ± 0.28	38.60 ± 0.23	1.16 ± 0.25	0.93 ± 0.06	2.24 ± 0.30	18.5 ± 0.07	29.7 ± 0.21	0.59 ± 0.12	0.55 ± 0.42
	Ultrasound treated sample	1st	16.00 ± 0.47	36.19 ± 0.02	38.61 ± 0.07	1.16 ± 0.63	0.93 ± 0.06	2.26 ± 0.07	17.7 ± 0.23	28.3 ± 0.53	0.76 ± 0.39	0.55 ± 0.06
		3rd	16.00 ± 0.01	36.20 ± 0.09	38.70 ± 0.47	1.16 ± 0.14	0.93 ± 0.11	2.26 ± 0.11	17.6 ± 0.47	29.0 ± 0.01	0.84 ± 0.21	0.55 ± 0.11
		5th	16.00 ± 0.14	36.20 ± 0.19	38.70 ± 0.63	1.16 ± 0.64	0.93 ± 0.01	2.26 ± 0.21	17.5 ± 0.32	25.8 ± 0.09	0.99 ± 0.01	0.55 ± 0.47
		7th	16.00 ± 0.23	36.20 ± 0.17	38.70 ± 0.53	1.16 ± 0.09	0.93 ± 0.25	2.26 ± 0.01	17.5 ± 0.63	24.8 ± 0.12	1.18 ± 0.38	0.55 ± 0.25
		9th	16.00 ± 0.32	36.20 ± 0.38	38.70 ± 0.21	1.16 ± 0.01	0.93 ± 0.23	2.26 ± 0.25	17.5 ± 0.19	24.4 ± 0.47	1.26 ± 0.14	0.55 ± 0.09

Every value is a mean of three determinations (*n* = 3) ± standard deviations. G/F: glucose to fructose ratio; G/W: glucose to water content ratio; HMF: hydroxymethylfurfural.

**Table 2:** Results of microbiological determination of honey sample.

Honey Variety		Month	Microorganism (cfu/g)				
			SPC	*Bacillus Cereus*	TC	Yeasts	Molds
Acacia	Control sample	1st	<10	-	-	<10	-
		3rd	<10	-	-	<10	-
		5th	<10	-	-	10	-
		7th	10	-	-	10	-
		9th	10	-	-	10	-
	Ultrasound treated sample	1st	-	-	-	-	-
		3rd	-	-	-	-	-
		5th	-	-	-	-	-
		7th	-	-	-	-	-
		9th	-	-	-	-	-
Raspberry	Control sample	1st	30	-	-	20	<10
		3rd	30	-	-	20	<10
		5th	30	-	-	20	<10
		7th	30	-	-	30	10
		9th	40	-	-	30	30
	Ultrasound treated sample	1st	<10	-	-	<10	<10
		3rd	<10	-	-	<10	<10
		5th	<10	-	-	<10	<10
		7th	10	-	-	10	<10
		9th	10	-	-	10	<10
Tillia	Control sample	1st	20	-	-	10	10
		3rd	20	-	-	10	10
		5th	20	-	-	20	20
		7th	30	-	-	20	30
		9th	30	-	-	30	40
	Ultrasound treated sample	1st	<10	-	-	-	10
		3rd	10	-	-	-	10
		5th	10	-	-	-	10
		7th	10	-	-	-	10
		9th	10	-	-	-	10
Polyfloral	Control sample	1st	<10	-	-	<10	20
		3rd	<10	-	-	20	20
		5th	<10	-	-	20	30
		7th	10	-	-	30	30
		9th	20	-	-	40	40
	Ultrasound treated sample	1st	<10	-	-	-	10
		3rd	<10	-	-	-	10
		5th	<10	-	-	-	10
		7th	<10	-	-	-	10
		9th	<10	-	-	-	10
Rapeseed	Control sample	1st	40		-	20	<10
		3rd	40	-	-	30	<10
		5th	40	-	-	30	<10
		7th	40	-	-	40	10
		9th	50	-	-	40	20
	Ultrasound treated sample	1st	20	-	-	-	<10
		3rd	20	-	-	-	<10
		5th	20	-	-	-	<10
		7th	20	-	-	-	<10
		9th	20	-	-	-	<10
Honeydew	Control sample	1st	<10	-	-	<10	<10
		3rd	<10	-	-	<10	<10
		5th	<10	-	-	<10	<10
		7th	<10	-	-	<20	10
		9th	<10	-	-	<20	10
	Ultrasound treated sample	1st	-	-	-	-	-
		3rd	-	-	-	-	-
		5th	-	-	-	-	-
		7th	-	-	-	-	-
		9th	-	-	-	-	-
Grassland	Control sample	1st	<10	-	-	<10	<10
		3rd	<10	-	-	<10	<10
		5th	<10	-	-	<10	<10
		7th	<10	-	-	<10	<10
		9th	<10	-	-	<20	<10
	Ultrasound treated sample	1st	-	-	-	-	-
		3rd	-	-	-	-	-
		5th	-	-	-	-	-
		7th	-	-	-	-	-
		9th	-	-	-	-	-

SPC: Standard Plate Count; TC: Total Coliforms; “-“: undetectable.

## Data Availability

Data available on request.

## Supplementary Materials

The following are available online at https://www.mdpi.com/article/10.3390/foods10040773/s1, Table S1: Color parameters of honey samples, Table S2: Texture parameters of honey samples, Table S3 (a–g): Pearson correlation.

## References

[1] (2002). Council Directive 2001/110/EC of 20 December 2001 relating to honey. Off. J. Eur. Commun..

[2] Kňazovická V., Gábor M., Miluchová M., Bobko M., Medo J. (2019). Diversity of bacteria in Slovak and foreign honey, with assessment of its physico-chemical quality and counts of cultivable microorganisms. J. Microbiol. Biotechnol. Food Sci..

[3] Pohl P., Stecka H., Sergiel I., Jamroz P. (2012). Different aspects of the elemental analysis of honey by flame atomic absorption and emission spectrometry: A review. Food Anal. Methods.

[4] Can Z., Yildiz O., Sahin H., Turumtay A.E., Silici S., Kolayli S. (2015). An investigation of Turkish honeys: Their physico-chemical properties, antioxidant capacities and phenolic profiles. Food Chem..

[5] Baloš M. Ž., Jakšić S., Popov N., Knežević S.V., Pelić D.L., Pelić M., Milanov D. (2019). Physicochemical characteristics of Serbian honeydew honey. Arch. Vet. Med..

[6] Laos K., Kirs E., Pall R., Martverk K. (2011). The crystallization behaviour of Estonian honeys. Agron. Res..

[7] Escuredo O., Dobre I., Fernández-Gonzálezs M., Seijo M.C. (2014). Contribution of botanical origin and sugar composition of honeys on the crystallization phenomenon. Food Chem..

[8] Ma Y., Zhang B., Li H., Li Y., Hu J., Li J., Wang H., Deng Z. (2017). Chemical and molecular dynamics analysis of crystallization properties of honey. Int. J. Food Prop..

[9] Amariei S., Norocel L., Scripcă L.A. (2020). An innovative method for preventing honey crystallization. Innov. Food Sci. Emerg. Technol..

[10] Dobre I., Georgescu I.A., Alexe P., Escuredo O., Seijo M.C. (2012). Rheological behavior of different honey types from Romania. Food Res. Int..

[11] Scripca L., Amariei S. (2018). Research on Honey Crystalization. Rev. De Chim..

[12] Živkov Baloš M., Jakšić S., Popov N., Mihaljev Ž., Ljubojević Pelić D. (2019). Comparative study of water content in honey produced in different years. Arch. Vet. Med..

[13] Tanleque-Alberto F., Juan-Borras M., Escriche I. (2019). Quality parameters, pollen and volatile profiles of honey from North and Central Mozambique. Food Chem..

[14] Braghini F., Biluca F.C., Gonzaga L.V., Vitali L., Costa A.C.O., Fett R. (2021). Effect thermal processing in the honey of Tetragonisca angustula: Profile physicochemical, individual phenolic compounds and antioxidant capacity. J. Apic. Res..

[15] Majid I., Nayik G.A., Nanda V. (2015). Ultrasonication and food technology: A review. Cogent Food Agric..

[16] Joseph A.K. (2017). Antibacterial activity of honey against bacteria isolated from wound at the Tamale teaching Hospital A Thesis submitted in partial fulfillment of the requirements for the degree of bachelor of science in the department of biomedical laboratory sciences. School of Allied Health Science.

[17] Chaikham P., Kemsawasd V., Apichartsrangkoon A. (2016). Effects of conventional and ultrasound treatments on physicochemical properties and antioxidant capacity of floral honeys from Northern Thailand. Food Biosci..

[18] Stojković M., Cvetković D., Savić A., Topalić-Trivunović L., Velemir A., Papuga S., Žabić M. (2020). Changes in the physicochemical, antioxidant and antibacterial properties of honeydew honey subjected to heat and ultrasound pretreatments. J. Food Sci. Technol..

[19] Mahnot N.K., Saikia S., Mahanta C.L. (2019). Quality characterization and effect of sonication time on bioactive properties of honey from North East India. J. Food Sci. Technol..

[20] Önür İ., Misra N.N., Barba F.J., Putnik P., Lorenzo J.M., Gökmen V., Alpas H. (2018). Effects of ultrasound and high pressure on physicochemical properties and HMF formation in Turkish honey types. J. Food Eng..

[21] Quintero-Lira A., Ángeles Santos A., Aguirre-Álvarez G., Reyes-Munguía A., Almaraz-Buendía I., Campos-Montiel R.G. (2017). Effects of liquefying crystallized honey by ultrasound on crystal size, 5-hydroxymethylfurfural, colour, phenolic compounds and antioxidant activity. Eur. Food Res. Technol..

[22] Phawatwiangnak K., Intipunya P. (2013). Melting of crystallized sunflower honey by high power ultrasonic method. Food Appl. Biosci. J..

[23] Janghu S., Bera M.B., Nanda V., Rawson A. (2017). Study on power ultrasound optimization and its comparison with conventional thermal processing for treatment of raw honey. J. Food Technol. Biotechnol..

[24] Basmaci I. (2010). Effect of Ultrasound and High Hydrostatic Pressure (HHP) on Liquefaction and Quality Parameters of Selected Honey Varieties. Master’s Thesis.

[25] Solis-Silva R., de Hidalgo A.D.E., Figueira A.C., Almaraz-Buendia I., Quintero-Lira A., Rodríguez O.D.R., Campos-Montiel R.G. (2017). Effect of ultrasound in bioactive compounds and antioxidant activity in during the storage of a monofloral honey. Book Proc..

[26] Akyol E., Güneşdoğdu M. (2019). The effect of heating the honey with bein-marie method and ultrasonic bath on honey crystallization. Turk. J. Agric. Food Sci. Technol..

[27] Chaikham P., Prangthip P. (2015). Alteration of antioxidative properties of longan flower-honey after high pressure, ultrasonic and thermal processing. J. Food Biosci..

[28] Chandrapala J., Oliver C., Kentish S., Ashokkumar M. (2012). Ultrasonics in food processing–Food quality assurance and food safety. J. Food Sci. Technol..

[29] Pingret D., Fabiano-Tixier A.S., Chemat F. (2013). Degradation during application of ultrasound in food processing: A review. Food Control.

[30] Mortas¸ M., Yazici F. (2013). Application of ultrasound technology to honey. Mellifera.

[31] Bogdanov S., Martin P., Lullmann C. (2002). Harmonised methods of the international honey commission. Swiss Bee Res. Cent. FamLiebefeld.

[32] Scripcă L.A., Norocel L., Amariei S. (2019). Comparison of Physicochemical, Microbiological Properties and Bioactive Compounds Content of Grassland Honey and other Floral Origin Honeys. Molecules.

[33] ***STAS 784-2009 Miere de albine.

[34] Kuntzler S.G., Costa J.A.V., Brizio A.P.D.R., Morais M.G. (2020). Development of a colorimetric pH indicator using nanofibers containing Spirulina sp. LEB 18. Food Chem..

[35] Semjon B., Marcinčáková D., Koréneková B., Bartkovský M., Nagy J., Turek P., Marcinčák S. (2020). Multiple factorial analysis of physicochemical and organoleptic properties of breast and thigh meat of broilers fed a diet supplemented with humic substances. Poult. Sci..

[36] (2004). CIE 015:2004 Colorimetry.

[37] Pascual-Maté A., Osés S.M., Marcazzan G.L., Gardini S., Fernández Muiño M.A., Sancho M.T. (2018). Sugar composition and sugar-related parameters of honeys from the northern Iberian Plateau. J. Food Comp. Anal..

[38] Codex Alimentarius Commission (2001). Draft Revised Standard for Honey (at Step 10 of the Codex Procedure).

[39] Viuda-Martos M., Ruiz-Navajas Y., Zaldivar-Cruz J.M., Kuri V., Fernández-López J., Carbonell-Barrachina Á.A., Pérez-Álvarez J. (2010). Aroma profile and physico-chemical properties of artisanal honey from Tabasco, Mexico. Int. J. Food Sci. Technol..

[40] Berk B. (2020). Determination of Honey Crystallization and Adulteration by Using Time Domain NMR Relaxometry. Master’s Thesis.

[41] Singh I., Singh S. (2018). Honey moisture reduction and its quality. J. Food Sci. Technol..

[42] Płowaś-Korus I., Masewicz Ł., Szwengiel A., Rachocki A., Baranowska H.M., Medycki W. (2018). A novel method of recognizing liquefied honey. Food Chem..

[43] Kek S.P., Chin N.L., Yusof Y.A., Tan S.W., Chua L.S. (2017). Classification of entomological origin of honey based on its physicochemical and antioxidant properties. Int. J. Food Prop..

[44] Brudzynski K., Miotto D., Kim L., Sjaarda C., Maldonado-Alvarez L., Fukś H. (2017). Active macromolecules of honey form colloidal particles essential for honey antibacterial activity and hydrogen peroxide production. Sci. Rep..

[45] Al-Jouri E., Daher-Hjaij N., Alkattea R., Alsayed Mahmoud K., Saffan A.M. (2017). Evaluation of changes in some physical and chemical properties of syrian honey, affecting honey crystallization due to the different geographical sites. Biol. Forum Int. J..

[46] Marghitaș L. Al., Dezmirean D., Moise A., Bobiș O., Laslo L., Bogdanov S. (2009). Physico-chemical and bioactive properties of different floral origin honeys from Romania. Food Chem..

[47] Abramovič H., Jamnik M., Burkan L., Kač M. (2008). Water activity and water content in Slovenian honeys. Food Control.

[48] Maldonado G.E., Navarro A.S., Yamul D.K. (2018). A comparative study of texture and rheology of Argentinian honeys from two regions. J. Texture Stud..

[49] Chirife J., Zamora M.C., Motto A. (2006). The correlation between water activity and % moisture in honey: Fundamental aspects and application to Argentine honeys. J. Food Eng..

[50] El Sohaimy S.A., Masry S.H.D., Shehata M.G. (2015). Physicochemical characteristics of honey from different origins. Ann. Agric. Sci..

[51] Chua L.S., Adnan N.A. (2014). Biochemical and nutritional components of selected honey samples. Acta Sci. Pol. Technol. Aliment..

[52] Mădaş N.M., Mărghitaş L.A., Dezmirean D.S., Bonta V., Bobiş O., Fauconnier M.L., Francis F., Haubruge E., Nguyen K.B. (2019). Volatile profile and physico-chemical analysis of acacia honey for geographical origin and nutritional value determination. Foods.

[53] Łuczycka D., Pentoś K., Wysoczański T. (2016). The influence of crystallization and temperature on electrical parameters of honey. Zesz. Probl. Postępów Nauk Rol..

[54] Chong K.Y., Chin N.L., Yusof Y.A. (2017). Thermosonication and optimization of stingless bee honey processing. J. Int. Food Sci. Technol..

[55] Aljohar H.I., Maher H.M., Albaqami J., Al-Mehaizie M., Orfali R., Orfali R., Alrubia S. (2018). Physical and chemical screening of honey samples available in the Saudi market: An important aspect in the authentication process and quality assessment. Saudi Pharmaceut. J..

[56] Alghamdi B.A., Alshumrani E.S., Saeed M.S.B., Rawas G.M., Alharthi N.T., Baeshen M.N., Helmi N.M., Alam M.Z., Suhail M. (2020). Analysis of sugar composition and pesticides using HPLC and GC–MS techniques in honey samples collected from Saudi Arabian markets. Saudi J. Biol. Sci..

[57] Gürbüz S., Çakıcı N., Mehmetoğlu S., Atmaca H., Demir T., Arıgül Apan M., Güney F. (2020). Physicochemical quality characteristics of Southeastern Anatolia honey, Turkey. Int. J. Anal. Chem..

[58] Homrani M., Escuredo O., Rodríguez-Flores M.S., Fatiha D., Mohammed B., Homrani A., Seijo M.C. (2020). Botanical Origin, Pollen Profile, and Physicochemical Properties of Algerian Honey from Different Bioclimatic Areas. Foods.

[59] Tomczyk M., Tarapatskyy M., Dżugan M. (2019). The influence of geographical origin on honey composition studied by Polish and Slovak honeys. Czech J. Food Sci..

[60] Zielińska S., Wesołowska M., Bilek M., Kaniuczak J., Dżugan M. (2020). The saccharide profile of Polish honeys depending on their botanical origin. J. Microbiol. Biotechnol. Food Sci..

[61] Geană E.I., Ciucure C.T., Costinel D., Ionete R.E. (2020). Evaluation of honey in terms of quality and authenticity based on the general physicochemical pattern, major sugar composition and δ13C signature. Food Control.

[62] Roby M.H., Abdelaliem Y.F., Esmail A.H.M., Mohdaly A.A., Ramadan M.F. (2020). Evaluation of Egyptian honeys and their floral origins: Phenolic compounds, antioxidant activities, and antimicrobial characteristics. Environ. Sci. Pollut. Res..

[63] Lazarević K.B., Andrić F., Trifković J., Tešić Ž., Milojković-Opsenica D. (2012). Characterisation of Serbian unifloral honeys according to their physicochemical parameters. Food Chem..

[64] Baloš M. Ž., Popov N., Vidaković S., Pelić D.L., Pelić M., Mihaljev Ž., Jakšić S. (2018). Electrical conductivity and acidity of honey. Arch. Vet. Med..

[65] Ratiu I.A., Al-Suod H., Bukowska M., Ligor M., Buszewski B. (2020). Correlation study of honey regarding their physicochemical properties and sugars and cyclitols content. Molecules.

[66] Kędzierska-Matysek M., Florek M., Wolanciuk A., Skałecki P. (2016). Effect of freezing and room temperatures storage for 18 months on quality of raw rapeseed honey (Brassica napus). J. Food Sci. Technol..

[67] Zarei M., Fazlara A., Alijani N. (2019). Evaluation of the changes in physicochemical and antioxidant properties of honey during storage. Functional Foods in Health and Disease..

[68] Nedić N., Gojak M., Zlatanović I., Rudonja N., Lazarević K., Dražić M., Gligorević K.B., Pajić M. (2020). Study of vacuum and freeze drying of bee honey. Therm. Sci..

[69] Soares S., Pinto D., Rodrigues F., Alves R.C., Oliveira M.B.P.P. (2017). Portuguese honeys from different geographical and botanical origins: A 4-year stability study regarding quality parameters and antioxidant activity. Molecules.

[70] Razali M.F., Fauzi N.A.M., Sulaiman A., Rahman N.A.A. (2019). Effect of high-pressure processing (hpp) on antioxidant, diastase activity and colour for Kelulut (stingless bee) honey. J. Technol..

[71] Sajid M., Yasmin T., Asad F., Qamer S. (2019). Changes in HMF content and diastase activity in honey after heating treatment. Pure Appl. Biol..

[72] Vranić D., Petronijević R., Stojanović J.Đ., Korićanac V., Milijašević J.B., Milijašević M. (2017). Physicochemical properties of honey from Serbia in the period 2014–2016. Proceedings of the IOP Conference Series: Earth and Environmental Science.

[73] Rojas M.L., Hellmeister Trevilin J., Duarte Augusto P.E. (2016). The ultrasound technology for modifying enzyme activity. Sci. Agropecu..

[74] Behzadnia A., Moosavi-Nasab M., Ojha S., Tiwari B.K. (2020). Exploitation of Ultrasound Technique for Enhancement of Microbial Metabolites Production. Molecules.

[75] Mawson R., Gamage M., Terefe N.S., Knoerzer K. (2011). Ultrasound in enzyme activation and inactivation. Ultrasound Technologies for Food and Bioprocessing.

[76] Patrignani M., Ciappini M.C., Tananaki C., Fagúndez G.A., Thrasyvoulou A., Lupano C.E. (2018). Correlations of sensory parameters with physicochemical characteristics of Argentinean honeys by multivariate statistical techniques. Int. J. Food Sci. Technol..

[77] Aypak S.Ü., İnci A., Bakırcı S., Fidan E.D., Soysal M. (2019). Comparision of the antioxidant activity and hydroxymethylfurfural (HMF) levels in honey taken from hives and markets. Gıda.

[78] Kędzierska-Matysek M., Florek M., Wolanciuk A., Skałecki P., Litwińczuk A. (2016). Characterisation of viscosity, colour, 5-hydroxymethylfurfural content and diastase activity in raw rape honey (Brassica napus) at different temperatures. J. Food Sci. Technol..

[79] Shapla U.M., Solayman M., Alam N., Khalil M.I., Gan S.H. (2018). 5-Hydroxymethylfurfural (HMF) levels in honey and other food products: Effects on bees and human health. Chem. Cent. J..

[80] Pravcová K., Mikysek T., Česlová L. (2020). Comparison of HPLC and electrochemical determination of 5-hydroxymethylfurfural in honey and mead samples. Sci. Pap. Univ. Pardubic. Ser. A Fac. Chem.Technol..

[81] Taylor M.J., Alabdrabalameer H.A., Skoulou V. (2019). Choosing physical, physicochemical and chemical methods of pre-treating lignocellulosic wastes to repurpose into solid fuels. Sustainability.

[82] Bizzi C.A., Santos D., Sieben T.C., Motta G.V., Mello P.A., Flores E.M.M. (2019). Furfural production from lignocellulosic biomass by ultrasound-assisted acid hydrolysis. Ultrason. Sonochemistry.

[83] Isla M.I., Craig A., Ordoñez R., Zampini C., Sayago J., Bedascarrasbure E., Alvarez A., Salomon V., Maldonado L. (2011). Physico chemical and bioactive properties of honeys from Northwestern Argentina. LWT Food Sci. Technol..

[84] El-Haskoury R., Kriaa W., Lyoussi B., Makni M. (2018). Ceratonia siliqua honeys from Morocco: Physicochemical properties, mineral contents, and antioxidant activities. J. Food Drug Anal..

[85] Zábrodská B., Vorlová L. (2015). Adulteration of honey and available methods for detection—A review. Acta Vet. Brno.

[86] Bertoncelj J., Doberšek U., Jamnik M., Golob T. (2007). Evaluation of the phenolic content, antioxidant activity and colour of Slovenian honey. Food Chem..

[87] Halagarda M., Groth S., Popek S., Rohn S., Pedan V. (2020). Antioxidant activity and phenolic profile of selected organic and conventional honeys from Poland. Antioxidants.

[88] Kuś P.M., Congiu F., Teper D., Sroka Z., Jerković I., Tuberoso C.I.G. (2014). Antioxidant activity, color characteristics, total phenol content and general HPLC fingerprints of six Polish unifloral honey types. LWT J. Food Sci. Technol..

[89] Stasiak D.M., Dolatowski Z.J. (2007). Effect of sonication on the crystallization of honeys. Pol. J. Food Nutr. Sci..

[90] Kabbani D., Sepulcre F., Wedekind J. (2011). Ultrasound-assisted liquefaction of rosemary honey: Influence on rheology and crystal content. J. Food Eng..

[91] Yikmiş S. (2020). Effect of ultrasound on different quality parameters of functional sirkencubin syrup. Food Sci. Technol..

[92] Starek A., Kobus Z., Sagan A., Chudzik B., Pawłat J., Kwiatkowski M., Terebun P., Andrejko D. (2021). Influence of ultrasound on selected microorganisms, chemical and structural changes in fresh tomato juice. Sci. Rep..

[93] Li J., Ding T., Liao X., Chen S., Ye X., Liu D. (2017). Synergetic effects of ultrasound and slightly acidic electrolyzed water against Staphylococcus aureus evaluated by flow cytometry and electron microscopy. Ultrason. Sonochem..

[94] Lee J.J., Eifert J.D., Jung S., Strawn L.K. (2018). Cavitation Bubbles Remove and Inactivate Listeria and Salmonella on the Surface of Fresh Roma Tomatoes and Cantaloupes. Front. Sustain. Food Syst..

[95] Zupanc M., Pandur Ž., Perdih T.S., Stopar D., Petkovšek M., Dular M. (2019). Effects of cavitation on different microorganisms: The current understanding of the mechanisms taking place behind the phenomenon. A review and proposals for further research. Ultrason. Sonochem..

[96] Chaven S. (2014). Honey, confectionery and bakery products. Food Saf. Manag..

