# Supplementary material for: The Use of Ultrasound for Preventing Honey Crystallization
Source: Foods. 2021 Apr 4;10(4):773. doi: 10.3390/foods10040773 (PMC8066198; doi:10.3390/foods10040773)
Supplement: Supplementary file 1 [file foods-10-00773-s001.pdf]

foods

Table S1. Supplement material – Color parameters of honey samples

Honey variety		Month	L*	a*	b	Croma	Hue angle, degrees	Yellow Index
Acacia	Control sample	1st	46.24±0.33	-0.83±0.13	16.34±0.38	4.65±0.46	-1.52±0.01	50.48±0.04
		3rd	46.21±0.14	-0.79±0.22	16.28±0.33	4.62±0.31	-1.52±0.12	50.33±0.14
		5th	46.16±0.09	-0.74±0.31	16.19±0.21	4.57±0.04	-1.53±0.16	50.10±0.12
		7th	46.12±0.53	-0.72±0.09	16.11±0.19	4.52±0.09	-1.53±0.07	49.90±0.18
		9th	46.09±0.17	-0.69±0.48	16.09±0.52	4.51±0.16	-1.53±0.15	49.87±0.33
	Ultrasound treated sample	1st	46.26±0.07	-0.83±0.16	16.35±0.25	4.66±0.04	-1.52±0.01	50.49±0.06
		3rd	46.23±0.09	-0.81±0.02	16.33±0.53	4.65±0.23	-1.52±0.06	50.46±0.11
		5th	46.20±0.46	-0.80±0.11	16.33±0.21	4.65±0.06	-1.52±0.05	50.49±0.17
		7th	46.18±0.47	-0.78±0.19	16.32±0.23	4.64±0.42	-1.52±0.02	50.48±0.08
		9th	46.15±0.19	-0.77±0.06	16.30±0.64	4.63±0.38	-1.52±0.01	50.45±0.21
Raspberry	Control sample	1st	28.02±0.25	4.31±0.46	14.86±0.29	3.85±0.11	1.28±0.01	75.76±0.13
		3rd	27.11±0.01	3.94±0.24	15.27±0.03	4.06±0.09	1.31±0.21	80.46±0.01
		5th	26.78±0.53	3.69±0.32	15.63±0.11	4.26±0.64	1.33±0.47	83.38±0.04
		7th	26.43±0.19	3.41±0.29	15.97±0.21	4.45±0.53	1.36±0.04	86.32±0.29
		9th	26.04±0.30	3.12±0.06	16.25±0.07	4.60±0.07	1.38±0.19	89.15±0.15
	Ultrasound treated sample	1st	28.34±0.29	4.33±0.16	14.84±0.47	3.84±0.09	1.28±0.09	74.80±0.33
		3rd	28.15±0.12	4.30±0.11	14.88±0.30	3.86±0.07	1.28±0.21	75.51±0.27
		5th	27.98±0.01	4.26±0.06	14.93±0.42	3.89±0.04	1.29±0.47	76.22±0.22
		7th	27.89±0.29	4.21±0.32	14.98±0.12	3.91±0.23	1.29±0.32	76.73±0.09
		9th	27.67±0.64	4.17±0.25	15.02±0.09	3.93±0.64	1.29±0.19	77.54±0.13
Tillia	Control sample	1st	31.97±0.30	-0.36±0.12	14.94±0.63	3.89±0.09	-1.55±0.16	66.76±0.16
		3rd	31.90±0.63	-0.49±0.14	15.23±0.53	4.04±0.06	-1.54±0.09	68.20±0.24
		5th	31.68±0.01	-0.73±0.01	15.47±0.64	4.17±0.01	-1.52±0.05	69.76±0.11
		7th	31.43±0.11	-0.99±0.15	15.84±0.12	4.37±0.63	-1.51±0.17	71.99±0.16
		9th	31.27±0.29	-1.21±0.17	16.22±0.14	4.59±0.12	-1.50±0.01	74.10±0.29
	Ultrasound treated sample	1st	31.99±0.30	-0.37±0.08	14.94±0.42	3.89±0.64	-1.55±0.01	66.71±0.24
		3rd	31.95±0.07	-0.39±0.01	14.98±0.07	3.91±0.07	-1.54±0.21	66.98±0.13
		5th	31.92±0.53	-0.46±0.07	15.08±0.23	3.96±0.09	-1.54±0.04	67.49±0.08
		7th	31.89±0.11	-0.52±0.03	15.15±0.63	4.00±0.30	-1.54±0.14	67.86±0.27
		9th	31.86±0.01	-0.55±0.11	15.21±0.01	4.03±0.06	-1.53±0.01	68.20±0.06
Polyfloral	Control sample	1st	39.65±0.25	4.53±0.29	13.99±0.09	3.41±0.11	1.25±0.32	50.40±0.19
		3rd	39.33±0.39	4.37±0.09	14.24±0.11	3.53±0.21	1.27±0.04	51.72±0.29
		5th	39.12±0.01	4.14±0.01	14.47±0.63	3.65±0.25	1.29±0.09	52.84±0.16
		7th	38.87±0.30	4.01±0.42	14.95±0.04	3.90±0.07	1.30±0.71	54.94±0.24
		9th	38.56±0.38	3.87±0.06	15.12±0.29	3.99±0.14	1.32±0.38	56.01±0.08
	Ultrasound treated sample	1st	39.79±0.38	4.54±0.12	13.95±0.21	3.39±0.06	1.25±0.04	50.08±0.13
		3rd	39.68±0.11	4.49±0.07	13.99±0.30	3.41±0.30	1.26±0.25	50.36±0.27
		5th	39.59±0.04	4.41±0.13	14.01±0.07	3.42±0.64	1.26±0.01	50.55±0.11
		7th	39.53±0.38	4.37±0.11	14.06±0.16	3.45±0.01	1.26±0.19	50.81±0.08
		9th	39.49±0.11	4.32±0.06	14.11±0.63	3.47±0.38	1.27±0.36	51.04±0.29

Table S1. Cont.

Honey variety		Month	L*	a*	b	Croma	Hue angle, degrees	Yellow Index
Rape	Control sample	1st	33.68±0.38	-2.19±0.01	18.61±0.21	6.04±0.63	-1.45±0.08	78.93±0.24
		3rd	31.28±0.22	-3.58±0.11	18.01±0.22	5.66±0.19	-1.37±0.01	82.25±0.13
		5th	29.83±0.04	-4.07±0.01	17.14±0.07	5.12±0.25	-1.34±0.05	82.08±0.16
		7th	27.58±0.63	-4.75±0.05	16.06±0.15	4.50±0.21	-1.28±0.06	83.18±0.04
		9th	26.06±0.07	-5.24±0.08	15.12±0.32	3.99±0.07	-1.24±0.01	83.88±0.19
	Ultrasound treated sample	1st	33.70±0.53	-2.20±0.01	18.60±0.29	6.07±0.13	-1.46±0.08	78.85±0.11
		3rd	32.48±0.63	-2.98±0.05	18.42±0.19	5.99±0.25	-1.46±0.06	81.02±0.27
		5th	31.54±0.21	-3.27±0.06	18.16±0.07	5.92±0.23	-1.45±0.05	82.26±0.08
		7th	30.62±0.47	-3.77±0.08	17.86±0.10	5.83±0.25	-1.45±0.01	83.33±0.29
		9th	29.24±0.53	-4.06±0.01	17.18±0.48	5.71±0.48	-1.46±0.01	83.94±0.24
Honeydew	Control sample	1st	21.08±0.04	7.14±0.25	10.24±0.21	1.83±0.32	0.96±0.06	69.39±0.04
		3rd	19.99±0.48	7.29±0.48	10.02±0.30	1.75±0.06	0.94±0.21	71.60±0.27
		5th	18.65±0.21	7.74±0.10	9.69±0.23	1.63±0.13	0.89±0.28	74.22±0.13
		7th	17.59±0.25	7.94±0.07	9.37±0.47	1.53±0.29	0.86±0.23	76.09±0.24
		9th	16.17±0.32	8.17±0.21	9.02±0.22	1.42±0.25	0.83±0.01	79.69±0.19
	Ultrasound treated sample	1st	21.17±0.30	7.09±0.10	10.22±0.21	1.82±0.12	0.96±0.42	68.96±0.11
		3rd	20.93±0.06	7.12±0.29	10.18±0.19	1.80±0.06	0.96±0.21	69.48±0.08
		5th	20.58±0.21	7.16±0.30	10.15±0.28	1.79±0.29	0.95±0.17	70.45±0.29
		7th	20.15±0.10	7.20±0.12	10.07±0.64	1.76±0.07	0.95±0.01	71.39±0.27
		9th	19.64±0.23	7.25±0.06	9.99±0.09	1.74±0.09	0.94±0.30	72.66±0.08
Grassland	Control sample	1st	34.56±0.47	2.11±0.53	12.59±0.48	2.76±0.25	1.40±0.53	52.04±0.11
		3rd	34.51±0.38	1.99±0.12	12.09±0.29	2.55±0.48	1.40±0.47	50.04±0.11
		5th	33.07±0.46	1.73±0.07	11.78±0.47	2.42±0.63	1.42±0.38	50.88±0.24
		7th	32.49±0.09	1.48±0.10	11.51±0.11	2.31±0.11	1.44±0.19	50.60±0.13
		9th	31.86±0.48	1.32±0.14	11.21±0.07	2.19±0.09	1.45±0.64	50.26±0.04
	Ultrasound treated sample	1st	34.62±0.13	2.16±0.32	12.63±0.63	2.78±0.63	1.40±0.38	52.11±0.19
		3rd	34.49±0.63	2.14±0.48	12.51±0.11	2.73±0.01	1.40±0.09	51.81±0.29
		5th	34.36±0.01	2.09±0.42	12.33±0.21	2.60±0.47	1.40±0.19	51.26±0.13
		7th	34.12±0.53	2.04±0.01	12.18±0.23	2.58±0.21	1.40±0.23	50.99±0.29
		9th	33.92±0.48	2.01±0.38	12.05±0.06	2.53±0.53	1.40±0.12	50.75±0.24

Every value is a mean of three determinations (n = 3) ± standard deviations; L* - degree of brightness (luminosity); a*, b* - color tonality.

Table S2. Supplement material – Texture parameters of honey samples

Honey variety		Month	Hardness	Springiness	Cohesiveness	Adhesiveness	Viscosity	Chewiness	Gumminess
Acacia	Control sample	1st	0.77±0.05	0.31±0.07	1.34±0.08	1.37±0.11	0.51±0.01	0.14±0.03	0.24±0.09
		3rd	0.79±0.01	0.31±0.03	1.34±0.11	1.36±0.03	0.55±0.12	0.14±0.09	0.25±0.07
		5th	0.81±0.08	0.29±0.12	1.35±0.01	1.34±0.12	0.58±0.08	0.16±0.07	0.26±0.03
		7th	0.82±0.09	0.28±0.03	1.37±0.07	1.33±0.08	0.62±0.03	0.17±0.08	0.26±0.05
		9th	0.83±0.07	0.26±0.04	1.38±0.12	1.31±0.09	0.65±0.04	0.18±0.03	0.28±0.14
	Ultrasound treated sample	1st	0.76±0.03	0.32±0.11	1.34±0.05	1.37±0.08	0.51±0.01	0.13±0.07	0.24±0.01
		3rd	0.77±0.01	0.33±0.09	1.34±0.11	1.38±0.01	0.51±0.12	0.13±0.12	0.24±0.03
		5th	0.77±0.05	0.33±0.07	1.35±0.01	1.39±0.11	0.52±0.07	0.14±0.03	0.24±0.07
		7th	0.78±0.09	0.32±0.05	1.35±0.11	1.39±0.07	0.53±0.04	0.14±0.08	0.24±0.05
		9th	0.78±0.07	0.32±0.03	1.36±0.12	1.39±0.03	0.54±0.03	0.14±0.01	0.25±0.11
Raspberry	Control sample	1st	0.92±0.08	0.59±0.14	0.89±0.03	1.33±0.05	0.54±0.12	0.68±0.05	0.82±0.09
		3rd	0.92±0.03	0.59±0.07	0.89±0.01	1.33±0.07	0.56±0.03	0.62±0.09	0.82±0.03
		5th	0.92±0.01	0.58±0.08	0.88±0.07	1.34±0.09	0.56±0.07	0.58±0.12	0.82±0.07
		7th	0.92±0.11	0.58±0.05	0.88±0.11	1.35±0.07	0.58±0.01	0.53±0.07	0.81±0.01
		9th	0.94±0.09	0.58±0.03	0.88±0.03	1.35±0.11	0.58±0.08	0.49±0.03	0.81±0.11
	Ultrasound treated sample	1st	0.92±0.07	0.59±0.07	0.89±0.09	1.35±0.07	0.54±0.07	0.68±0.09	0.82±0.05
		3rd	0.92±0.03	0.58±0.12	0.89±0.07	1.33±0.01	0.54±0.12	0.68±0.12	0.82±0.09
		5th	0.92±0.05	0.58±0.01	0.88±0.08	1.34±0.12	0.54±0.03	0.68±0.08	0.81±0.07
		7th	0.92±0.11	0.58±0.04	0.88±0.01	1.35±0.05	0.54±0.12	0.68±0.11	0.81±0.01
		9th	0.91±0.01	0.58±0.07	0.88±0.03	1.37±0.12	0.54±0.01	0.67±0.07	0.81±0.08
Tillia	Control sample	1st	0.82±0.12	0.45±0.09	2.35±0.11	1.18±0.11	0.60±0.14	0.82±0.05	1.92±0.03
		3rd	0.96±0.08	0.45±0.05	2.40±0.07	1.16±0.03	0.60±0.08	0.81±0.12	1.88±0.05
		5th	0.98±0.07	0.44±0.07	2.45±0.03	1.16±0.07	0.62±0.09	0.80±0.14	1.85±0.09
		7th	0.99±0.09	0.42±0.01	2.46±0.12	1.16±0.01	0.62±0.07	0.78±0.12	1.84±0.03
		9th	1.00±0.03	0.40±0.08	2.48±0.11	1.14±0.12	0.63±0.12	0.77±0.03	1.79±0.07
	Ultrasound treated sample	1st	0.80±0.14	0.43±0.07	2.40±0.01	1.28±0.09	0.60±0.03	0.82±0.07	1.92±0.11
		3rd	0.80±0.01	0.43±0.12	2.40±0.05	1.28±0.03	0.60±0.01	0.81±0.12	1.92±0.08
		5th	0.80±0.07	0.42±0.03	2.40±0.03	1.29±0.01	0.60±0.12	0.81±0.05	1.89±0.01
		7th	0.82±0.12	0.42±0.08	2.50±0.08	1.29±0.11	0.60±0.09	0.81±0.12	1.88±0.09
		9th	0.82±0.09	0.42±0.07	2.50±0.03	1.29±0.08	0.62±0.05	0.81±0.07	1.88±0.07

Table S2. Cont.

Honey variety		Month	Hardness	Springiness	Cohesiveness	Adhesiveness	Viscosity	Chewiness	Gumminess
Polyfloral	Control sample	1st	0.82±0.12	0.60±0.01	0.87±0.09	1.23±0.07	0.77±0.03	0.72±0.12	0.70±0.01
		3rd	1.00±0.03	0.52±0.04	0.97±0.14	1.11±0.11	0.94±0.08	0.65±0.01	0.59±0.08
		5th	1.27±0.11	0.40±0.09	1.25±0.11	0.98±0.05	1.28±0.07	0.59±0.07	0.57±0.05
		7th	1.36±0.05	0.39±0.07	1.47±0.08	0.82±0.12	1.36±0.04	0.54±0.09	0.54±0.09
		9th	1.49±0.08	0.39±0.03	1.68±0.01	0.70±0.09	1.48±0.03	0.49±0.12	0.51±0.12
	Ultrasound treated sample	1st	0.82±0.09	0.60±0.07	0.86±0.11	1.22±0.03	0.74±0.09	0.72±0.01	0.70±0.03
		3rd	0.98±0.12	0.59±0.12	0.87±0.09	1.16±0.08	0.74±0.07	0.72±0.09	0.67±0.07
		5th	0.99±0.07	0.58±0.03	0.89±0.03	1.12±0.04	0.76±0.12	0.71±0.04	0.66±0.09
		7th	1.01±0.05	0.56±0.01	0.89±0.12	1.12±0.09	0.76±0.05	0.71±0.08	0.66±0.03
		9th	1.04±0.04	0.56±0.08	0.89±0.05	1.10±0.14	0.76±0.03	0.71±0.03	0.64±0.05
Rape	Control sample	1st	0.84±0.03	0.78±0.09	1.06±0.07	3.16±0.03	0.56±0.12	0.78±0.07	0.89±0.14
		3rd	1.67±0.11	0.71±0.07	1.08±0.01	2.81±0.09	0.69±0.12	0.84±0.09	1.98±0.11
		5th	2.12±0.01	0.66±0.08	1.12±0.11	2.35±0.04	0.83±0.07	0.92±0.05	2.29±0.09
		7th	2.46±0.07	0.56±0.03	1.18±0.09	1.96±0.09	0.98±0.01	1.04±0.04	2.65±0.03
		9th	2.83±0.08	0.41±0.07	1.29±0.12	1.35±0.01	1.27±0.04	1.28±0.07	3.02±0.08
	Ultrasound treated sample	1st	0.82±0.09	0.77±0.08	1.05±0.08	3.18±0.07	0.58±0.03	0.77±0.01	0.91±0.01
		3rd	0.97±0.01	0.76±0.05	1.25±0.03	3.20±0.11	0.64±0.04	0.75±0.08	1.70±0.14
		5th	1.04±0.09	0.73±0.09	1.34±0.07	3.19±0.12	0.67±0.11	0.69±0.12	2.13±0.11
		7th	1.16±0.14	0.70±0.01	1.56±0.01	3.03±0.03	0.70±0.14	0.66±0.05	3.01±0.07
		9th	1.31±0.03	0.68±0.08	1.87±0.12	2.79±0.01	0.73±0.03	0.64±0.07	4.48±0.01
Honeydew	Control sample	1st	0.93±0.12	0.55±0.08	0.93±0.09	1.46±0.07	0.57±0.07	0.32±0.08	0.82±0.03
		3rd	0.99±0.07	0.51±0.03	0.96±0.07	1.40±0.08	0.65±0.09	0.45±0.12	0.71±0.08
		5th	1.08±0.08	0.43±0.14	0.99±0.07	1.36±0.11	0.79±0.11	0.56±0.08	0.64±0.07
		7th	1.26±0.11	0.38±0.04	1.09±0.03	1.32±0.04	0.93±0.07	0.71±0.11	0.60±0.09
		9th	1.43±0.05	0.36±0.03	1.17±0.04	1.28±0.05	1.36±0.05	0.84±0.01	0.49±0.05
	Ultrasound treated sample	1st	0.94±0.09	0.55±0.08	0.93±0.08	1.45±0.07	0.52±0.03	0.31±0.07	0.82±0.03
		3rd	0.95±0.07	0.59±0.05	0.96±0.12	1.43±0.04	0.53±0.08	0.29±0.08	0.84±0.01
		5th	0.96±0.03	0.63±0.07	0.96±0.01	1.43±0.09	0.56±0.04	0.28±0.04	0.84±0.08
		7th	1.08±0.01	0.69±0.03	0.98±0.03	1.41±0.05	0.62±0.01	0.28±0.09	0.85±0.07
		9th	1.08±0.08	0.71±0.11	0.98±0.04	1.40±0.07	0.64±0.03	0.27±0.07	0.85±0.03
Grassland	Control sample	1st	1.04±0.07	0.31±0.12	0.91±0.09	1.62±0.11	0.60±0.09	0.33±0.12	0.94±0.01
		3rd	1.06±0.11	0.31±0.09	0.93±0.07	1.60±0.09	0.65±0.11	0.32±0.01	0.94±0.11
		5th	1.14±0.09	0.29±0.07	0.96±0.04	1.58±0.07	0.68±0.07	0.32±0.04	0.94±0.09
		7th	1.20±0.07	0.28±0.01	0.98±0.08	1.57±0.05	0.70±0.09	0.32±0.05	0.92±0.08
		9th	1.24±0.01	0.26±0.11	1.01±0.01	1.52±0.11	0.75±0.12	0.31±0.11	0.92±0.07
	Ultrasound treated sample	1st	1.00±0.11	0.31±0.09	0.91±0.04	1.62±0.04	0.60±0.14	0.33±0.08	0.94±0.05
		3rd	1.00±0.03	0.32±0.12	0.93±0.03	1.64±0.09	0.60±0.04	0.32±0.03	0.98±0.03
		5th	1.04±0.14	0.32±0.03	0.96±0.05	1.68±0.04	0.60±0.05	0.32±0.07	1.02±0.01
		7th	1.04±0.07	0.33±0.08	0.98±0.07	1.69±0.07	0.60±0.08	0.32±0.04	1.04±0.09
		9th	1.02±0.05	0.33±0.07	1.01±0.11	1.70±0.03	0.62±0.01	0.32±0.09	1.06±0.11

Every value is a mean of three determinations (n = 3) ± standard deviations.

Table S3. Supplement material - Pearson correlation

Table S3a. Pearson correlation for physicochemical parameters of acacia honey

Variables	W	Glucose	Fructose	Sucrose	G/F	G/W	ID	HMF	Wa	L*	a*	b*	C	H	YI	Ha	Sp	Co	Ad	Vi	Ch	Gu
W	**1**																					
Glucose	0.262	**1**																				
Fructose	-0.319	-0.249	**1**																			
Sucrose	0.442	0.620	-0.350	**1**																		
G/F	-0.931	-0.161	0.493	-0.470	**1**																	
G/W	-0.978	-0.094	0.393	-0.371	0.961	**1**																
ID	0.959	0.085	-0.309	0.398	-0.960	-0.976	**1**															
HMF	-0.918	-0.135	0.531	-0.461	0.945	0.950	-0.955	**1**														
Wa	0.816	0.207	-0.758	0.500	-0.940	-0.869	0.839	-0.915	**1**													
L*	-0.221	-0.611	0.583	-0.184	0.234	0.173	-0.040	0.113	-0.387	**1**												
a*	0.419	0.983	-0.321	0.640	-0.330	-0.264	0.248	-0.285	0.360	-0.663	**1**											
b*	-0.637	-0.563	0.607	-0.264	0.597	0.594	-0.481	0.531	-0.668	0.874	-0.678	**1**										
C	-0.636	-0.562	0.607	-0.263	0.598	0.594	-0.480	0.531	-0.669	0.874	-0.677	1.000	**1**									
H	0.408	0.987	-0.280	0.667	-0.313	-0.247	0.242	-0.275	0.333	-0.606	0.997	-0.624	-0.623	**1**								
YI	-0.711	-0.531	0.590	-0.274	0.661	0.671	-0.567	0.611	-0.710	0.813	-0.657	0.993	0.993	-0.606	**1**							
Ha	0.581	0.558	-0.694	0.264	-0.599	-0.553	0.456	-0.527	0.704	-0.889	0.671	-0.983	-0.984	0.615	-0.970	**1**						
Sp	-0.757	-0.625	0.572	-0.502	0.751	0.703	-0.637	0.656	-0.775	0.770	-0.752	0.945	0.945	-0.713	0.952	-0.935	**1**					
Co	0.287	0.706	-0.407	0.302	-0.211	-0.196	0.056	-0.086	0.302	-0.946	0.746	-0.844	-0.843	0.702	-0.789	0.810	-0.770	**1**				
Ad	-0.863	-0.562	0.509	-0.434	0.809	0.808	-0.755	0.771	-0.793	0.635	-0.700	0.915	0.915	-0.666	0.948	-0.888	0.963	-0.643	**1**			
Vi	0.604	0.641	-0.588	0.299	-0.571	-0.545	0.440	-0.481	0.640	-0.900	0.746	-0.989	-0.989	0.696	-0.974	0.981	-0.948	0.878	-0.899	**1**		
Ch	0.615	0.622	-0.631	0.402	-0.596	-0.566	0.464	-0.517	0.681	-0.885	0.729	-0.973	-0.973	0.680	-0.958	0.965	-0.957	0.868	-0.891	0.972	**1**	
Gu	0.592	0.735	-0.554	0.395	-0.576	-0.513	0.438	-0.483	0.632	-0.858	0.830	-0.938	-0.938	0.789	-0.922	0.940	-0.937	0.834	-0.896	0.962	0.930	**1**

W-Water Content, Glucose, Fructose, Sucrose, G/F, G/W, ID- Diastase Index, HMF- Hydroxymethylfurfural, L*, a*, b*, C-Chroma, H-Hue Angle, YI- Yellow Index, Ha-Hardness, Sp-Springiness, Co-Cohesiveness, Ad-Adhesiveness, Vi-Viscosity, Ch-Chewiness, Gu-Gumminess.

Table S3. Supplement material - Pearson correlation

Table S3b. Pearson correlation for physicochemical parameters of raspberry honey

Variables	W	Glucose	Fructose	Sucrose	G/F	G/W	ID	HMF	Wa	L*	a*	b*	C	H	YI	Ha	Sp	Co	Ad	Vi	Ch	Gu
W	**1**																					
Glucose	-0.171	**1**																				
Fructose	0.852	-0.299	**1**																			
Sucrose	0.853	-0.333	0.896	**1**																		
G/F	-0.753	0.643	-0.923	-0.854	**1**																	
G/W	-1.000	0.178	-0.854	-0.855	0.757	**1**																
ID	-0.876	0.306	-0.945	-0.985	0.881	0.878	**1**															
HMF	-0.162	1.000	-0.291	-0.327	0.636	0.169	0.298	**1**														
Wa	-0.171	1.000	-0.299	-0.333	0.643	0.178	0.306	1.000	**1**													
L*	0.466	-0.617	0.593	0.747	-0.725	-0.469	-0.694	-0.614	-0.617	**1**												
a*	0.436	-0.701	0.577	0.689	-0.746	-0.440	-0.651	-0.698	-0.701	0.981	**1**											
b*	-0.434	0.679	-0.579	-0.687	0.739	0.438	0.651	0.676	0.679	-0.980	-0.999	**1**										
C	-0.432	0.685	-0.578	-0.685	0.740	0.436	0.649	0.682	0.685	-0.978	-0.999	1.000	**1**									
H	-0.439	0.686	-0.579	-0.692	0.741	0.442	0.654	0.683	0.686	-0.984	-1.000	0.999	0.999	**1**								
YI	-0.449	0.665	-0.589	-0.715	0.740	0.453	0.672	0.662	0.665	-0.992	-0.997	0.997	0.996	0.998	**1**							
Ha	-0.317	0.905	-0.494	-0.429	0.761	0.323	0.461	0.902	0.905	-0.572	-0.669	0.654	0.660	0.657	0.631	**1**						
Sp	-0.583	-0.218	-0.307	-0.218	0.159	0.581	0.264	-0.224	-0.218	0.331	0.353	-0.369	-0.369	-0.357	-0.352	-0.094	**1**					
Co	-0.302	-0.272	-0.209	0.000	0.058	0.300	0.101	-0.276	-0.272	0.501	0.494	-0.506	-0.505	-0.499	-0.503	-0.117	0.802	**1**				
Ad	0.351	0.167	0.555	0.333	-0.378	-0.351	-0.451	0.171	0.167	-0.147	-0.178	0.179	0.180	0.176	0.166	-0.167	-0.400	-0.612	**1**			
Vi	-0.526	0.583	-0.672	-0.750	0.775	0.529	0.730	0.579	0.583	-0.961	-0.970	0.971	0.970	0.972	0.972	0.607	-0.218	-0.357	0.062	**1**		
Ch	0.492	-0.681	0.634	0.720	-0.784	-0.496	-0.692	-0.677	-0.681	0.974	0.996	-0.996	-0.996	-0.996	-0.993	-0.670	0.298	0.432	-0.130	-0.981	**1**	
Gu	-0.512	-0.333	-0.384	-0.200	0.174	0.510	0.300	-0.339	-0.333	0.316	0.345	-0.345	-0.347	-0.341	-0.335	-0.143	0.655	0.816	-0.667	-0.250	0.279	**1**

W-Water Content, Glucose, Fructose, Sucrose, G/F, G/W, ID- Diastase Index, HMF- Hydroxymethylfurfural, L*, a*, b*, C-Chroma, H-Hue Angle, YI- Yellow Index, Ha-Hardness, Sp-Springiness, Co-Cohesiveness, Ad-Adhesiveness, Vi-Viscosity, Ch-Chewiness, Gu-Gumminess.

foods

MDPI

Table S3. Supplement material - Pearson correlation

Table S3c. Pearson correlation for physicochemical parameters of tillia honey

Variables	W	Glucose	Fructose	Sucrose	G/F	G/W	ID	HMF	Wa	L*	a*	b*	C	H	YI	Ha	Sp	Co	Ad	Vi	Ch	Gu
W	**1**																					
Glucose	0.729	**1**																				
Fructose	-0.383	-0.647	**1**																			
Sucrose	0.064	-0.072	-0.167	**1**																		
G/F	0.667	0.960	-0.834	0.009	**1**																	
G/W	-0.998	-0.686	0.351	-0.076	-0.625	**1**																
ID	0.842	0.936	-0.736	-0.066	0.946	-0.813	**1**															
HMF	-0.888	-0.888	0.672	-0.116	-0.888	0.867	-0.955	**1**														
Wa	0.156	0.264	-0.408	0.408	0.341	-0.143	0.244	-0.245	**1**													
L*	-0.194	-0.388	0.749	-0.515	-0.555	0.173	-0.425	0.378	-0.731	**1**												
a*	-0.150	-0.335	0.746	-0.521	-0.516	0.132	-0.380	0.338	-0.741	0.997	**1**											
b*	0.170	0.367	-0.781	0.501	0.552	-0.150	0.414	-0.367	0.759	-0.992	-0.996	**1**										
C	0.173	0.369	-0.777	0.504	0.552	-0.153	0.414	-0.369	0.765	-0.992	-0.996	1.000	**1**									
H	0.142	0.328	-0.750	0.517	0.512	-0.124	0.375	-0.331	0.727	-0.996	-1.000	0.995	0.995	**1**								
YI	0.177	0.373	-0.773	0.506	0.553	-0.157	0.417	-0.371	0.757	-0.995	-0.998	1.000	1.000	0.996	**1**							
Ha	0.647	0.716	-0.912	0.132	0.852	-0.626	0.851	-0.770	0.442	-0.741	-0.723	0.752	0.750	0.724	0.749	**1**						
Sp	0.468	0.312	0.028	-0.583	0.215	-0.468	0.398	-0.373	-0.635	0.602	0.629	-0.596	-0.599	-0.626	-0.600	0.072	**1**					
Co	-0.477	-0.535	-0.228	0.438	-0.303	0.458	-0.393	0.392	0.317	-0.521	-0.574	0.548	0.544	0.584	0.540	0.102	-0.677	**1**				
Ad	-0.738	-0.805	0.871	-0.070	-0.902	0.713	-0.920	0.845	-0.434	0.658	0.632	-0.667	-0.667	-0.630	-0.666	-0.983	-0.175	0.064	**1**			
Vi	0.096	0.163	-0.611	0.611	0.342	-0.090	0.222	-0.213	0.616	-0.869	-0.879	0.865	0.864	0.882	0.867	0.609	-0.551	0.644	-0.507	**1**		
Ch	-0.080	-0.361	0.732	-0.470	-0.529	0.054	-0.368	0.297	-0.726	0.986	0.984	-0.981	-0.981	-0.983	-0.984	-0.685	0.645	-0.524	0.601	-0.823	**1**	
Gu	-0.076	-0.262	0.770	-0.406	-0.472	0.059	-0.325	0.270	-0.739	0.945	0.965	-0.971	-0.970	-0.967	-0.966	-0.716	0.596	-0.636	0.615	-0.859	0.933	**1**

W-Water Content, Glucose, Fructose, Sucrose, G/F, G/W, ID- Diastase Index, HMF- Hydroxymethylfurfural, L*, a*, b*, C-Chroma, H-Hue Angle, YI- Yellow Index, Ha-Hardness, Sp-Springiness, Co-Cohesiveness, Ad-Adhesiveness, Vi-Viscosity, Ch-Chewiness, Gu-Gumminess.

Table S3. Supplement material - Pearson correlation

Table S3d. Pearson correlation for physicochemical parameters of polyfloral honey

Variables	W	Glucose	Fructose	Sucrose	G/F	G/W	ID	HMF	Wa	L*	a*	b*	C	H	YI	Ha	Sp	Co	Ad	Vi	Ch	Gu
W	**1**																					
Glucose	0.156	**1**																				
Fructose	0.156	0.333	**1**																			
Sucrose	-0.116	0.954	0.318	**1**																		
G/F	0.024	0.675	-0.471	0.644	**1**																	
G/W	-0.999	-0.122	-0.146	0.149	0.000	**1**																
ID	0.102	-0.944	-0.281	-0.982	-0.663	-0.135	**1**															
HMF	-0.084	0.877	0.251	0.908	0.624	0.115	-0.968	**1**														
Wa	0.064	0.816	0.408	0.779	0.444	-0.035	-0.757	0.696	**1**													
L*	0.344	0.679	0.711	0.599	0.079	-0.322	-0.541	0.468	0.851	**1**												
a*	0.388	0.562	0.674	0.480	-0.002	-0.371	-0.410	0.331	0.787	0.981	**1**											
b*	-0.282	-0.659	-0.688	-0.609	-0.077	0.261	0.551	-0.492	-0.824	-0.984	-0.968	**1**										
C	-0.281	-0.656	-0.693	-0.607	-0.071	0.260	0.549	-0.491	-0.820	-0.983	-0.968	1.000	**1**									
H	-0.356	-0.603	-0.676	-0.530	-0.035	0.337	0.464	-0.390	-0.811	-0.989	-0.997	0.985	0.985	**1**								
YI	-0.297	-0.664	-0.701	-0.607	-0.073	0.276	0.549	-0.487	-0.831	-0.990	-0.974	0.999	0.999	0.989	**1**							
Ha	-0.438	-0.519	-0.648	-0.421	0.022	0.422	0.374	-0.326	-0.779	-0.963	-0.987	0.948	0.947	0.982	0.954	**1**						
Sp	0.299	0.692	0.504	0.633	0.253	-0.276	-0.583	0.514	0.900	0.945	0.948	-0.935	-0.932	-0.953	-0.938	-0.946	**1**					
Co	-0.278	-0.652	-0.728	-0.615	-0.040	0.257	0.567	-0.518	-0.806	-0.975	-0.962	0.990	0.990	0.977	0.990	0.949	-0.936	**1**				
Ad	0.197	0.992	0.412	0.939	0.605	-0.163	-0.921	0.852	0.859	0.766	0.660	-0.747	-0.745	-0.698	-0.753	-0.620	0.768	-0.741	**1**			
Vi	-0.255	-0.738	-0.619	-0.700	-0.206	0.231	0.659	-0.602	-0.891	-0.964	-0.945	0.966	0.965	0.960	0.968	0.936	-0.980	0.977	-0.813	**1**		
Ch	0.234	0.712	0.679	0.671	0.134	-0.211	-0.626	0.568	0.887	0.983	0.959	-0.986	-0.985	-0.975	-0.988	-0.944	0.962	-0.988	0.793	-0.991	**1**	
Gu	0.289	0.659	0.596	0.572	0.150	-0.267	-0.525	0.458	0.916	0.971	0.954	-0.943	-0.940	-0.960	-0.951	-0.950	0.954	-0.921	0.742	-0.942	0.960	**1**

W-Water Content, Glucose, Fructose, Sucrose, G/F, G/W, ID- Diastase Index, HMF- Hydroxymethylfurfural, L*, a*, b*, C-Chroma, H-Hue Angle, YI- Yellow Index, Ha-Hardness, Sp-Springiness, Co-Cohesiveness, Ad-Adhesiveness, Vi-Viscosity, Ch-Chewiness, Gu-Gumminess.

Table S3. Supplement material - Pearson correlation

Table S3e. Pearson correlation for physicochemical parameters of rape honey

Variables	W	Glucose	Fructose	Sucrose	G/F	G/W	ID	HMF	Wa	L*	a*	b*	C	H	YI	Ha	Sp	Co	Ad	Vi	Ch	Gu
W	**1**																					
Glucose	0.512	**1**																				
Fructose	0.896	0.333	**1**																			
Sucrose	0.128	0.111	0.333	**1**																		
G/F	-0.889	-0.306	-1.000	-0.333	**1**																	
G/W	-1.000	-0.510	-0.895	-0.130	0.888	**1**																
ID	0.879	0.745	0.756	0.351	-0.740	-0.879	**1**															
HMF	-0.932	-0.423	-0.977	-0.360	0.973	0.932	-0.845	**1**														
Wa	0.512	1.000	0.333	0.111	-0.306	-0.510	0.745	-0.423	**1**													
L*	-0.283	0.191	-0.386	0.433	0.396	0.281	0.140	0.243	0.191	**1**												
a*	-0.278	0.159	-0.376	0.502	0.385	0.275	0.133	0.226	0.159	0.987	**1**											
b*	-0.410	0.102	-0.480	0.332	0.488	0.408	0.010	0.365	0.102	0.975	0.937	**1**										
C	-0.596	-0.110	-0.617	0.274	0.619	0.594	-0.220	0.530	-0.110	0.915	0.881	0.971	**1**									
H	0.755	0.266	0.747	-0.239	-0.746	-0.753	0.427	-0.686	0.266	-0.824	-0.808	-0.890	-0.968	**1**								
YI	-0.173	-0.411	-0.002	-0.585	-0.011	0.176	-0.489	0.182	-0.411	-0.780	-0.839	-0.623	-0.486	0.388	**1**							
Ha	0.651	0.104	0.678	-0.334	-0.682	-0.649	0.272	-0.588	0.104	-0.905	-0.900	-0.938	-0.975	0.979	0.544	**1**						
Sp	-0.437	-0.012	-0.483	0.322	0.488	0.434	-0.047	0.383	-0.012	0.944	0.905	0.983	0.972	-0.904	-0.561	-0.928	**1**					
Co	-0.953	-0.553	-0.940	-0.357	0.933	0.953	-0.922	0.985	-0.553	0.148	0.136	0.279	0.471	-0.645	0.280	-0.525	0.309	**1**				
Ad	-0.595	-0.049	-0.631	0.256	0.635	0.593	-0.200	0.546	-0.049	0.913	0.875	0.973	0.993	-0.960	-0.477	-0.974	0.973	0.474	**1**			
Vi	0.473	0.057	0.494	-0.334	-0.497	-0.471	0.090	-0.397	0.057	-0.935	-0.901	-0.977	-0.979	0.918	0.540	0.938	-0.995	-0.333	-0.976	**1**		
Ch	0.786	0.350	0.720	-0.101	-0.716	-0.785	0.499	-0.697	0.350	-0.699	-0.652	-0.820	-0.921	0.949	0.146	0.885	-0.868	-0.677	-0.919	0.887	**1**	
Gu	-0.654	-0.596	-0.542	-0.613	0.529	0.656	-0.884	0.681	-0.596	-0.498	-0.526	-0.340	-0.134	-0.052	0.809	0.119	-0.290	0.762	-0.135	0.261	-0.203	**1**

W-Water Content, Glucose, Fructose, Sucrose, G/F, G/W, ID- Diastase Index, HMF- Hydroxymethylfurfural, L*, a*, b*, C-Chroma, H-Hue Angle, YI- Yellow Index, Ha-Hardness, Sp-Springiness, Co-Cohesiveness, Ad-Adhesiveness, Vi-Viscosity, Ch-Chewiness, Gu-Gumminess.

Table S3. Supplement material - Pearson correlation

Table S3f. Pearson correlation for physicochemical parameters of honeydew honey

Variables	W	Glucose	Fructose	Sucrose	G/F	G/W	ID	HMF	Wa	L*	a*	b*	C	H	YI	Ha	Sp	Co	Ad	Vi	Ch	Gu
W	**1**																					
Glucose	0.697	**1**																				
Fructose	-0.697	-1.000	**1**																			
Sucrose	-0.100	-0.333	0.333	**1**																		
G/F	0.697	1.000	-1.000	-0.333	**1**																	
G/W	-1.000	-0.689	0.689	0.097	-0.689	**1**																
ID	0.769	0.693	-0.693	-0.148	0.693	-0.771	**1**															
HMF	-0.823	-0.897	0.897	0.272	-0.897	0.820	-0.932	**1**														
Wa	0.244	0.816	-0.816	-0.408	0.816	-0.237	0.544	-0.696	**1**													
L*	0.000	-0.574	0.574	0.729	-0.574	-0.008	-0.157	0.365	-0.779	**1**												
a*	0.142	0.664	-0.664	-0.684	0.664	-0.136	0.322	-0.508	0.826	-0.979	**1**											
b*	-0.055	-0.584	0.584	0.751	-0.584	0.049	-0.228	0.416	-0.778	0.994	-0.987	**1**										
C	-0.050	-0.586	0.586	0.741	-0.586	0.043	-0.225	0.415	-0.783	0.995	-0.988	1.000	**1**									
H	-0.106	-0.630	0.630	0.720	-0.630	0.100	-0.282	0.470	-0.805	0.989	-0.997	0.996	0.997	**1**								
YI	-0.021	0.557	-0.557	-0.750	0.557	0.028	0.116	-0.334	0.758	-0.998	0.967	-0.987	-0.988	-0.979	**1**							
Ha	-0.052	0.443	-0.443	-0.782	0.443	0.058	0.021	-0.232	0.639	-0.969	0.924	-0.968	-0.967	-0.947	0.972	**1**						
Sp	-0.526	-0.814	0.814	0.520	-0.814	0.523	-0.785	0.864	-0.849	0.722	-0.832	0.773	0.772	0.809	-0.688	-0.617	**1**					
Co	-0.021	0.457	-0.457	-0.807	0.457	0.026	0.112	-0.289	0.649	-0.961	0.928	-0.972	-0.969	-0.952	0.959	0.984	-0.672	**1**				
Ad	0.058	-0.547	0.547	0.693	-0.547	-0.065	-0.152	0.346	-0.804	0.992	-0.971	0.987	0.988	0.981	-0.986	-0.957	0.721	-0.954	**1**			
Vi	0.122	0.582	-0.582	-0.872	0.582	-0.116	0.229	-0.424	0.716	-0.965	0.947	-0.974	-0.971	-0.964	0.968	0.961	-0.742	0.967	-0.943	**1**		
Ch	0.277	0.743	-0.743	-0.699	0.743	-0.271	0.485	-0.647	0.875	-0.935	0.974	-0.959	-0.958	-0.970	0.917	0.874	-0.915	0.899	-0.931	0.934	**1**	
Gu	-0.286	-0.768	0.768	0.698	-0.768	0.281	-0.519	0.677	-0.908	0.920	-0.961	0.939	0.939	0.954	-0.904	-0.840	0.922	-0.862	0.917	-0.921	-0.993	**1**

W-Water Content, Glucose, Fructose, Sucrose, G/F, G/W, ID- Diastase Index, HMF- Hydroxymethylfurfural, L*, a*, b*, C-Chroma, H-Hue Angle, YI- Yellow Index, Ha-Hardness, Sp-Springiness, Co-Cohesiveness, Ad-Adhesiveness, Vi-Viscosity, Ch-Chewiness, Gu-Gumminess.

foods MDPI

Table S3. Supplement material - Pearson correlation

Table S3g. Pearson correlation for physicochemical parameters of grassland honey

Variables	W	Glucose	Fructose	Sucrose	G/F	G/W	ID	HMF	Wa	L*	a*	b*	C	H	YI	Ha	Sp	Co	Ad	Vi	Ch	Gu
W	**1**																					
Glucose	0.250	**1**																				
Fructose	-0.660	0.258	**1**																			
Sucrose	-0.500	-0.125	0.330	**1**																		
G/F	0.688	-0.192	-0.998	-0.344	**1**																	
G/W	-1.000	-0.222	0.673	0.500	-0.699	**1**																
ID	0.599	-0.067	-0.811	-0.300	0.819	-0.605	**1**															
HMF	-0.648	0.068	0.892	0.297	-0.901	0.655	-0.969	**1**														
Wa	-0.500	-0.125	0.330	1.000	-0.344	0.500	-0.300	0.297	**1**													
L*	-0.571	-0.339	0.511	0.694	-0.542	0.564	-0.409	0.440	0.694	**1**												
a*	-0.704	-0.345	0.601	0.695	-0.635	0.699	-0.493	0.535	0.695	0.984	**1**											
b*	-0.695	-0.485	0.523	0.666	-0.565	0.686	-0.378	0.439	0.666	0.955	0.975	**1**										
C	-0.693	-0.494	0.518	0.656	-0.561	0.683	-0.371	0.434	0.656	0.951	0.972	1.000	**1**									
H	0.701	0.287	-0.617	-0.711	0.647	-0.697	0.526	-0.560	-0.711	-0.982	-0.996	-0.953	-0.948	**1**								
YI	-0.739	-0.635	0.376	0.393	-0.426	0.725	-0.186	0.293	0.393	0.539	0.632	0.765	0.772	-0.573	**1**							
Ha	0.711	0.346	-0.643	-0.664	0.677	-0.705	0.544	-0.608	-0.664	-0.962	-0.985	-0.956	-0.953	0.982	-0.626	**1**						
Sp	-0.693	-0.069	0.786	0.711	-0.803	0.696	-0.743	0.779	0.711	0.876	0.909	0.819	0.811	-0.932	0.426	-0.928	**1**					
Co	0.212	0.566	0.146	-0.495	-0.108	-0.197	-0.342	0.306	-0.495	-0.675	-0.618	-0.716	-0.719	0.577	-0.566	0.537	-0.275	**1**				
Ad	-0.724	0.014	0.875	0.629	-0.888	0.730	-0.891	0.905	0.629	0.727	0.790	0.693	0.685	-0.817	0.392	-0.820	0.954	-0.039	**1**			
Vi	0.760	0.304	-0.715	-0.722	0.748	-0.757	0.622	-0.668	-0.722	-0.931	-0.971	-0.949	-0.945	0.967	-0.675	0.965	-0.942	0.495	-0.879	**1**		
Ch	-0.500	-0.750	0.048	0.750	-0.102	0.481	-0.155	0.153	0.750	0.689	0.693	0.767	0.767	-0.665	0.685	-0.673	0.520	-0.707	0.410	-0.684	**1**	
Gu	-0.650	0.232	0.912	0.371	-0.910	0.661	-0.954	0.966	0.371	0.484	0.568	0.435	0.427	-0.607	0.186	-0.622	0.824	0.289	0.934	-0.675	0.093	**1**

W-Water Content, Glucose, Fructose, Sucrose, G/F, G/W, ID- Diastase Index, HMF- Hydroxymethylfurfural, L*, a*, b*, C-Chroma, H-Hue Angle, YI- Yellow Index, Ha-Hardness, Sp-Springiness, Co-Cohesiveness, Ad-Adhesiveness, Vi-Viscosity, Ch-Chewiness, Gu-Gumminess.
